# Advances in omics research on peanut response to biotic stresses

**DOI:** 10.3389/fpls.2023.1101994

**Published:** 2023-05-22

**Authors:** Ruihua Huang, Hongqing Li, Caiji Gao, Weichang Yu, Shengchun Zhang

**Affiliations:** ^1^ Guangdong Key Laboratory of Biotechnology for Plant Development, College of Life Sciences, South China Normal University, Guangzhou, China; ^2^ Guangdong Key Laboratory of Plant Epigenetics, College of Life Sciences and Oceanography, Shenzhen University, Shenzhen, China; ^3^ Liaoning Peanut Research Institute, Liaoning Academy of Agricultural Sciences, Fuxing, China; ^4^ China Good Crop Company (Shenzhen) Limited, Shenzhen, China

**Keywords:** Arachis, omics, pest, pathogen, fungi, virus, bacterial

## Abstract

Peanut growth, development, and eventual production are constrained by biotic and abiotic stresses resulting in serious economic losses. To understand the response and tolerance mechanism of peanut to biotic and abiotic stresses, high-throughput Omics approaches have been applied in peanut research. Integrated Omics approaches are essential for elucidating the temporal and spatial changes that occur in peanut facing different stresses. The integration of functional genomics with other Omics highlights the relationships between peanut genomes and phenotypes under specific stress conditions. In this review, we focus on research on peanut biotic stresses. Here we review the primary types of biotic stresses that threaten sustainable peanut production, the multi-Omics technologies for peanut research and breeding, and the recent advances in various peanut Omics under biotic stresses, including genomics, transcriptomics, proteomics, metabolomics, miRNAomics, epigenomics and phenomics, for identification of biotic stress-related genes, proteins, metabolites and their networks as well as the development of potential traits. We also discuss the challenges, opportunities, and future directions for peanut Omics under biotic stresses, aiming sustainable food production. The Omics knowledge is instrumental for improving peanut tolerance to cope with various biotic stresses and for meeting the food demands of the exponentially growing global population.

## 1 Introduction

*Arachis hypogaea* (peanut or groundnut) is among the most important oil and food legumes with annual production of ~46 million tons (http://www.fao.org/faostat/en/#home). It is cultivated in more than 100 countries around the world in tropical and subtropical regions, and is the principal source of digestible protein, cooking oil and vitamins in development and developing regions of Asia, Africa and America for fighting malnutrition and ensuring food security (Arya et al., 2015). Productivity levels of peanut in most of the developing countries have remained low due to several production constraints which include biotic and abiotic stresses. Breeding new cultivars to improve productivity is the best way to meet the needs of the producers, consumers and industry. As an allotetraploid species in the *Arachis* genus, peanut has extremely low genetic diversity because most of the other species in the genus are diploid (Bertioli et al., 2019). Peanut is particularly susceptible to a number of pest and pathogens due in part to the lack of gene exchange with its diploid wild ancestors that have resistance genes (Bertioli et al., 2016; Moretzsohn et al., 2013). The limited genetic diversity and the tetraploid complexity of cultivated gene pool is a barrier and challenge to create cultivars with broad resistance, excellent quality and high yield (Pandey et al., 2020). On the other hand, diploid wild relatives (*Arachis* spp.) with a larger genetic diversity evolving in a variety of habitats and biotic challenges are significant sources of resistance genes and a rich source of novel alleles that can be introduced into the cultivated species by unconventional method (Leal-Bertioli et al., 2009). Therefore, there is an urgent need to exploit gene resources in diploid species by using Omics methods.

Plant genome research has facilitated gene discovery and gene functional elucidation. With Omics, scientists can manage the intricate global biological systems based on advances in Omics technology (Mochida and Shinozaki, 2010). Recent advances in DNA sequencing technology have promoted the rapid development of science and made any other new applications beyond genome sequencing possible (Lister et al., 2009). Particularly, the emergence of next-generation sequencing makes whole-genome resequencing for variant discovery, transcriptional regulatory networks analysis, RNA sequencing analysis (RNA-seq) for transcriptome and noncoding RNAome, quantitative detection analysis (Chip-seq) for epigenome dynamics and DNA-protein interactions become viable applications (Lister et al., 2009). Other techniques, including interactomic analysis for protein-protein interactions, hormonomic analysis for plant hormone signaling, and metabolomic analysis of metabolic products, have been developed (Kojima et al., 2009; Saito and Matsuda, 2010). The omics technologies will help researchers to mine and screen specific genes involved in crop improvement. In addition, integrated network analysis reveals molecular connections between genes and metabolites, boots our understanding the relationships between phenotypic and genotype (Shinozaki and Sakakibara, 2009; Vadivel, 2015; Kumar et al., 2017). Over the past few decades, advances in genomics, transcriptomics, metabolomics, and proteomic analysis with the development of cutting-edge technologies have greatly facilitated the increase in the study of molecular aspects of peanut-biotic factor interactions. Therefore, different omics-based studies have attempted to decipher the molecular pathways that contribute to crop defenses against diseases and pests. In this review, we mainly retrospect the studies on the basic of vary Omics analyses concentrating mainly on those with relevant data on peanut defense responses and resistance to biotic stresses including insect pests, pathogen and bacteria.

## 2 Biotic stresses on peanut

### 2.1 Insect pests of peanut

In the semi-arid tropical regions, peanut is a significant crop and is a key component of the diets of both developed and emerging nations. Despite having a high potential for output, farmer’s fields typically yield very little due to insect pests and diseases pressure. The peanut crop is infringed by a large number of insects, which lead to disastrous consequences ranging from incidental feeding to almost whole plant destruction and finally yield loss (Wightman and Rao, 1994). According to Stalker and Campbell (1983), peanut is harmed by more than 350 kinds of insects, the most harmful of which are root-knot nematodes, *Aphis craccivora*, *Helicoverpa armigera*, and *Spodoptera litura* (**Table 1**).

**Table 1:** Impact of insect pests on peanut.

Name	Distribution	Symptom	Severity	Yield loss	Reference
Root-Knot Nematode	US、Africa and Asia	reduced growth and withering	most damaging plant-parasitic nematodes	annual billion dollar	Mota et al., 2018
*Aphis craccivora Koch*	worldwide	wilt, become yellow or brown, and eventually die	serious	around 20%	Blackman and Eastop, 2007
*Helicoverpa armigera*	Asia, Africa, southern Europe, and Australia	Feeding on plant’s flowering and fruiting bodies	destructive	over $US 2 billion annually	McGahan et al., 1991; Sharma, 2005; Tay et al., 2013
*Spodoptera litura*	Asia	severely defoliating	destructive	35–55%	Prasad and Gowda, 2006; Srinivasa et al., 2012

The root-knot nematode (RKN) *Meloidogyne arenaria* is a significant danger to peanut yield particularly in India, China, and the United States (Dong et al., 2008). The RKNs are obligate endoparasites of the *Meloidogyne* genus, with about 100 species described (Decraemer and Hunt, 2006), and the most destructive plant-parasitic nematodes worldwide (Jones et al., 2013), which can infest almost all cultivated plant species (Trudgill and Blok, 2001). The four most common RKN species causing most yield losses in crops are *Meloidogyne incognita*, *M. arenaria*, *M. javanica*, and *M. hapla* (Agrios, 2005). Plants infected by nematodes exhibit symptoms like reduced growth, withering, as well as increased sensitivity to other infections (Mota et al., 2018).

*Aphis craccivora* is an important group of insects with worldwide distribution. Most aphid species comprise a group of closely related populations which may have genetic divergence so that they could be considered as host races, nascent or sister species (or subspecies) (Blackman and Eastop, 2007). Aphid makes approximately 20% peanut yield loss, and causes damage on peanut from seedling to whole mature green plants (Blackman and Eastop, 2007). The Aphid causes both direct and indirect harm to peanut by removing the sap, causing irritation and toxicity, depositing honeydew, growing sooty mold, and spreading the rosette virus, e.g. at least seven viruses utilized Aphid as their vector to damage groundnuts, of which the *Peanut Stripe Virus* (PStV) and the *Groundnut Rosette Virus* (GRV) are the most significant (Blount et al., 2002). When Aphid infestation is severe, the plants may begin to wilt, become yellow or brown, and eventually die and peanut yields are significantly decreased (Blount et al., 2002).

*Helicoverpa armigera*, one of the most destructive agricultural pests, is thought to cost the US economy $2 billion annually (Tay et al., 2013). Asia, Africa, southern Europe, and Australia all have a significant population of *H. armigera* (Sharma, 2005). More than 200 plant species are impacted, including peanut (Pratissoli et al., 2015). Peanut yield is substantially impacted by *H. armigera*. The direct feeding behavior of *H. armigera* larvae on the plant’s flowering and fruiting bodies is one of the main explanations for a significant decline in agricultural productivity (McGahan et al., 1991).

*Spodoptera litura* is one of the most destructive species that larvae eat voraciously on leaves, severely defoliating the plant and only leaving the midrib veins, which can result in yield losses of 35–55% (Srinivasa et al., 2012). *S. litura* causes maximum damage at the stages during flowering and fruiting (Prasad and Gowda, 2006).

### 2.2 Microbial pathogen on peanut

#### 2.2.1 Fungi

Peanut growth and development is threatened by a variety of biotic stresses, of which the four fungal diseases leaf spot, rust, stem rot and *Aspergillus flavus* are predominate (**Table 2**).

**Table 2:** Impact of microbial pathogen on peanut.

Name	Distribution	Symptom	Severity	Yield loss	Reference
Leaf spot	worldwide	brown lesions around with a yellow ring (ELS),dark brown to black spots(LLS)	destructive disease	up to 70%	Tshilenge-Lukanda et al., 2012; Grichar et al., 1998
Rust	worldwide	reduced seed size, and low seed oil content	major fungal diseases	up to 70%	Subrahmanyam et al., 1993
Stem rot	widespread	Feeding on plant’s flowering and fruiting bodies	destructivedisease	80% yield loss	Thiessen and Woodward, 2012; Mehan et al., 1994; Punja, 1985
*Aspergillus flavus*	globally	induce aflatoxins	The major yield limiting biotic stress	Does not reduce yield directly	Krishna et al., 2015

Leaf spot includes the *Cercospora arachidicola*-caused early leaf spot (ELS) and the *Phaeoisariopsis personata*-caused late leaf spot (LLS). Both leaf spot diseases can occur on the leaves, petioles, stems, and pegs of peanut and produce lesions up to 1 centimeter in diameter (McDonald et al., 1985). Leaves infected with ELS generally show brown lesions around with a yellow ring on the upper side (Tshilenge-Lukanda et al., 2012), while LLS fungal disease usually exhibit dark brown to black spots (Tshilenge-Lukanda et al., 2012). ELS and LLS are destructive fungal diseases of cultivated peanut, causing yield losses of up to 70% under favorable conditions in the United States and around the world (Anco et al., 2020).

Rust, caused by *Puccinia arachidis* Speg. (Subrahmanyam et al., 1993), is another major fungal diseases restricting peanut yield in countries with warm, tropical climates, with losses as high as 50% reported in India (Varshney et al., 2014). Due to the tendency of rust-infected leaves to stay attached to the plant and the pathogen’s short life cycle, the disease can spread quickly and prodigiously. More seriously, rust-infected peanut reduce agricultural productivity, affects the seed oil quality, the haulm and the odder yield (Leal-Bertioli et al., 2015).

Stem rot is the deadliest disease in peanuts and produce markedly yields loss to peanut (Punja, 1985). Four mycelial compatibility groups (MCG) *S. rolfsii* were found among a total of 132 isolates from peanut fields in Ibaraki (Japan) and many isolates were clonal (Okabe and Matsumoto, 2000). This disease is widespread in peanut-growing areas (Thiessen and Woodward, 2012), and caused by *Sclerotium rolfsii* with thick, white hyphae that resemble silk in growth (Ma et al., 2022). Peanut infected with *S. rolfsii* generally exhibits the dark-brown lesions on the stem at soil surface or below the soil surface, followed by gradually yellowing and wilting of leaves (Termorshuizen, 2007). Peanut infect with *S. rolfsii* can produce rot on stem, peg and pod, and up to 80% yield loss (Mehan et al., 1994). Pessimistically, *S. rolfsii* is hard to control because sclerotia derived from *S. rolfsii* overwinter in the soil and attack peanut in the following season (Mayee et al., 1988; Le et al., 2012). After being infected with *S. rolfsii*, peanut plants may experience branch withering and perhaps complete plant wilting (Mayee et al., 1988; Le et al., 2012).

*Aspergillus flavus* fungus can produce Aflatoxin that threatens to the peanut industry (Krishna et al., 2015). Aflatoxin contaminated peanuts affect human and animal health when consumed (Pittet, 1998; Kew, 2013). Agonizingly, peanut pods and seeds can be infected and Aflatoxin is produced before harvest as well as during the steps of drying, storing, and transportation after harvest (Torres et al., 2014).

#### 2.2.2 Viruses

Peanuts are infected by various viruses, including *tomato spotted wilt virus* (TSWV), *cucumber mosaic virus* (CMV), *peanut stripe virus* (PStV), *peanut stunt virus* (PSV), *peanut bud necrosis virus* (PBNV), *peanut mottle virus* (PeMoV), *peanut ringspot virus* (PRSV) in the growth and development (**Table 3**).

**Table 3:** Impact of Viruses and Bacterial on peanut.

Name	Distribution	Symptom	Severity	Yield loss	Reference
Tomato Spotted Wilt Virus	southeastern United States	stunting phenotype	destructive disease	annual $12.3 million	Garcia et al., 2000; Culbreath et al., 2003; Riley et al., 2011
Stripe Virus	Asian country and United States	stripes, light mottle, and blotches	most prevalent plant-infecting viruses	around 20%	Choi et al., 2001; Singh et al., 2009; Middleton and Saleh, 1988
Bacterial wilt	globally	wilting and die	the most devastating diseases	10–100%	Mansfield et al., 2012; Genin, 2010; Salanoubat et al., 2002; Jiang et al., 2017

TSWV, a propagative and single-stranded RNA virus, is one of the most important pathogenic virus that attacks peanut in the southeastern United States (Culbreath and Srinivasan, 2011; Culbreath, et al., 2003; Garcia et al., 2000). TSWV is transmitted by at least 10 thrips species with a sustained and reproductive manner (Pappu et al., 2009; Riley et al., 2011), of which *Frankliniella fusca* and *F. occidentalis* are predominant (Riley et al., 2011). Peanut attacked by TSWV generally exhibit stunting phenotype particularly when TSWV infects peanut plant at an early stage of growth and development (Culbreath et al., 2003). Beyond that, peanut infected by TSWV also exhibits chlorosis, necrosis or ring spots in peanut leaves (Culbreath et al., 2003). It was reported that TSWV disease alone is estimated to cost US $12.3 million annually loss (Riley et al., 2011).

PStV is one of the most prevalent plant-infecting viruses, a member of the genus Potyvirus, and one of the largest groups of viruses that infect plants (Singh et al., 2009). PStV viruses have a 350-kD polyprotein that is translated by a single open reading frame, which are roughly 10 kb in length and carry a single positive-strand RNA (Urcuqui-Inchima et al., 2001; Wei et al., 2010). PStV is one of the most universal distributed peanut viruses limiting peanut yield by losing around 20%. A number of nations, including China, the US, the Philippines, Thailand, Indonesia, Malaysia, and Korea have reported PStV (Xu et al., 1983; Demski and Lovell, 1985; Saleh et al., 1989; Choi et al., 2001; Choi et al., 2006). PStV can be spread by aphids in a non-sustained manner. In addition, PStV was 10–100% prevalent in the fields and 1–50% in peanut seeds (Chen et al., 1990; Xu et al., 1991; Bi et al., 1999; Xu, 2002). The principal infection source in the field is the infected peanut seeds. On peanut, PStV can induce a number of symptoms, including stripes, light mottle, and blotches that is occasionally encircled by necrotic or chlorotic ringspots (Middleton and Saleh, 1988).

#### 2.2.3 Bacterial

Many bacterial diseases occur in peanuts grown in tropical and subtropical areas because of the warm and wet weather, of which bacterial wilt is predominant (Jiang et al., 2017).

Peanut bacterial wilt, caused by the soil-borne bacterium *Ralstonia solanacearum*, is one of the most devastating diseases in peanut (Salanoubat et al., 2002). *R. solanacearum* ranked second among the top 10 pathogenic bacteria in plant pathology because it spread worldwide and could survive many years in soils (Hayward, 1991; Mansfield et al., 2012). *R. solanacearum* attacks peanut generally through the root system and then spreads to the aboveground parts through the vascular system. If the bacteria breed up to high levels, the plant will show signs of wilting and die (Genin, 2010). In addition, Bacterial wilt disease can lead to 10–30% yield losses and 50–100% in severe circumstances (Jiang et al., 2017) (**Table 3**).

## 3 Importance and types of omics approaches for peanut science

**Figure 1:**
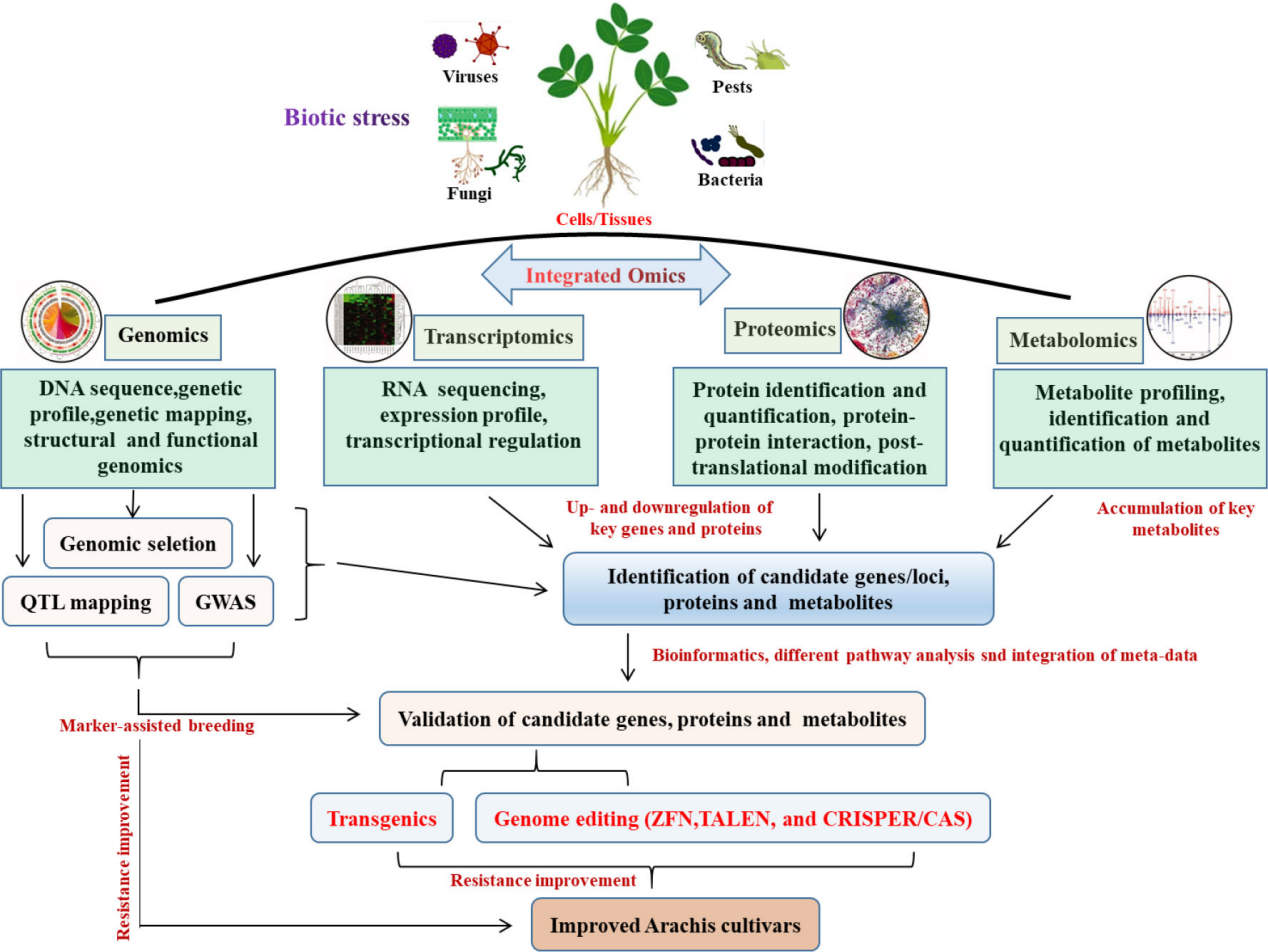
Flowchart for improvement of peanut abiotic stress resistance using Omics technologies.

The advancement of biotechnology to address plant productivity and stress tolerance has been sparked by the emergence of contemporary genetic engineering methodologies and high throughput biological research tools. Plant biotechnology combined with Omics has the potential to solve a number of issues that currently hinder agriculture, such as diseases and pests, pressures from the environment and climate change (Pérez-Clemente et al., 2013). Omics include but are not limited to genomics, transcriptomics, proteomics, epigenetics, metabolomics, miRNAomics, epigenomics and phenomics (Haas et al., 2017), all were used to improve the peanut cultivars (**Figure 1**). Genomics-assisted breeding (GAB) has demonstrated great potential for improving peanut varieties. High-quality genome assembly and well-annotated genome are very crucial for GAB. The succeed genome sequence assemblies of wild diploid progenitors, wild tetraploid and both the subspecies of cultivated tetraploids (Bertioli et al., 2016; Bertioli et al., 2019; Zhuang et al., 2019), providing a cornerstone for functional genomics and peanut improvement. Based on the availability of reference genome for both the diploid progenitors, genome-wide simple sequence repeat (SSR) markers were discovered (Zhao et al., 2017). Moreover, whole-genome resequencing (WGRS) of mapping populations has facilitated development of high-density single nucleotide polymorphism (SNP)–based genetic map and genome-wide SNP genotyping array, which were developed for fine mapping and candidate gene discovery for disease resistance in peanut (Clevenger et al., 2017; Pandey et al., 2017c; Agarwal et al., 2018). Although marker-assisted selection approaches have been used to develop superior peanut lines, technological advancements in sequencing and high-throughput genotyping can enhance genetic diversity and forward generation and genomic selection, as well as faster candidate gene discovery in the peanut breeding program (Varshney et al., 2019). For example, RNA sequencing (RNA-seq), which belongs to the transcriptomic approach, can improve the genome annotation and gene discovery especially for the genes which encodes for proteins and non-coding RNAs. Understanding the full metabolic networks involving genes, transcripts, proteins, and metabolites in biological systems is currently crucial because it is extremely difficult to succeed with the strategy of expression of a few single genes in peanut. In this regard, it becomes important to conduct a comprehensive analysis using functional genomics tools such as transcriptomics, proteomics, and metabolomics to characterize plant-pathogen interactions in order to unveil the genetic and metabolic responses of a specific plant species to infection (Pandey et al., 2020).

## 4 Omics advances in understanding peanut responses to biotic stress

### 4.1 Peanut responses to pests

#### 4.1.1 Root-knot nematodes

Most peanut cultivars are susceptible to the root-knot nematode (RKN) *M. arenaria*, while the wild diploid *Arachis* species exhibit high resistance (Holbrook and Stalker, 2003). Thus, many gene(s)/QTLs sources linked to RKN resistance were identified in different wild *Arachis* germplasm (**Table 4**). The interspecific *Arachis* hybrid TxAG-6 ([*A. batizocoi* × (*A. cardenasii* × *A. diogoi*)]^4x^) was the source of ELS and LLS resistance and the donor parent to introgress resistance into commercial cultivar. In order to improve peanut resistance to RKN, gene segments from wild TxAG-6 were introduced into peanut cultivars through an interspecific hybridization backcrossing scheme, and the root-knot nematode-resistant varieties, COAN and NemaTAM were developed with marker-assisted backcrossing is in the USA (Garcia et al., 1995; Simpson and Starr, 2001; Stalker et al., 2002; Simpson et al., 2003). Previously, the RKN resistance of the cultivated peanut is derived from introgression of a large segment on chromosome A09 from the wild species *A. cardenasii* (Nagy et al., 2010). *A. stenosperma* has high potential in peanut breeding as it owns strong RKN resistance (Ballén-Taborda et al., 2019). Three quantitative trait loci (QTL) were genetically mapped to strongly influence nematode root galling and egg production by using 93 recombinant inbred lines (RILs) developed from a cross between *A. duranensis* and *A. stenosperma* (Leal-Bertioli et al., 2016). Two loci controlling the resistance on chromosomes A02 and A09 have been validated in cultivar peanut to reduce nematode reproduction by up to 98%, and the large-effect QTL on A02 is enriched for genes encoding TIR-NBS-LRR proteins (Ballén-Taborda et al., 2019; Ballén-Taborda et al., 2021; Ballén-Taborda et al., 2022).

**Table 4:** QTL associated with Peanut responses to Root-knot nematodes.

	Location	Population	Reference
Root-knot nematodes	chromosomes 02, 04 and 09	*A. duranensis* × *A. stenosperma*	Bertioli et al., 2016
	chromosomes A02 and A09	(*A.batizocoi *× *A.stenorperma*) ×*A.hypogaea*	Ballén-Taborda et al., 2019; Ballén-Taborda et al., 2021; Ballén-Taborda et al., 2022
	chromosomes A09	*A.cardnesii*	Nagy et al., 2010

To understand the molecular components underlying RKN resistance, researchers took advantage of genomics and transcriptomics to demonstrated that wild *Arachis* species *A. stenosperma* harbors high levels of resistance to RKN infection through the onset of hypersensitive response (HR), which is usually caused by resistance genes (R) (Proite et al., 2010; Dang et al., 2013; Guimaraes et al., 2015). As we all know, the majority of plant R genes are the NBS-LRR type genes (Meyers et al., 1999). Transcriptome analysis of two peanut wild relatives, *A. stenosperma* representing the highly RKN resistant and *A. duranensis* as the moderately susceptible, during early stages of RKN infection, found that resistance genes against root-knot nematode infection were NBS-LRR class of plant disease resistance (R) genes. Two decades ago, 78 NBS-LRR coding sequences with unknown functions were identified in wild *Arachis* species *A. stenosperma* by resistance gene analogues (RGAs) targeting degenerate primers in the NBS domain (Bertioli et al., 2003). Furthermore, over 300 representative genes segmented into four NBS-LRR family types were isolated from the genome-wide analysis in the wild peanut species (Bertioli et al., 2016; Song et al., 2017). Similarly, hundreds of RGAs were identified from several peanut cultivars (Yuksel et al., 2005; Ratnaparkhe et al., 2011; Wang et al., 2012). Suppression subtractive hybridization (SSH) revealed that pathogenesis related (PR) protein, patatin-like protein and universal stress related protein (USPs) genes, which related to the resistance operative against invading nematodes, were expressed in the early stages of RKN-infected NemaTAM roots (Tirumalaraju et al., 2011). In addition, seven genes including one gene encoding a resistance protein MG13, were differentially expressed in RKN-infected wild *Arachis* (Morgante et al., 2013). Comparative genomics combining with differential gene expression analysis in 22 plant species including peanuts revealed the conserved immune response genes triggered by RKN infection. The core genes include plant defense and secondary metabolite production (Mota et al., 2020). In addition, genes involved in hormone signaling and secondary metabolites production may be involved in RKN resistance (Mota et al., 2018). Consistently, genes engaged in salicylic and jasmonic acids signaling pathways as well as genes in auxin balance regulation were found in the transcriptome analysis of RKN-resistant *Arachis* genotypes (Guimaraes et al., 2015). These results suggest the role of phytohormones in root-knot nematode resistance.

In addition to transcriptomics and genomics, metabolomics and miRNAomics are also making important contributions to peanut RKN research. The miRNAomics with whole-transcriptome RNA-seq revealed that 430 mRNAs, 111 miRNAs, 4453 lncRNAs, and 123 circRNAs were differentially expressed upon RKN infection, among which a total number of 10 lncRNAs, 4 circRNAs, 5 miRNAs, and 13 mRNAs involved in the oxidation reduction process and biological metabolism processes in RKN infected peanuts (Xu et al., 2022). Furthermore, proteome combining with transcriptome analysis identified differentially expressed proteins and genes during root-knot nematode infection (Martins et al., 2020). Most of the differentially expressed proteins are related to plant responses to pathogens. And the plant defense related genes encoding the ADH (alcohol dehydrogenase), CCR1 (cinnamoyl-CoA reductase 1), ENO (enolase), eIF5A (eukaryotic translation initiation factor 5A) and MLP34 (MLP-like protein 34) were found during peanut RKN infection, and the AsMLP34 was considered as a candidate in peanut RKN resistance (Martins et al., 2020).

#### 4.1.2 Aphis craccivora

To prevent *Aphis craccivora* infestation in peanut, resistance lines were first identified using phenomics (Padgham et al., 1990). Genomic analysis by Merwe et al. (2001) revealed that *Aphis craccivora* resistance was regulated by a single recessive gene. DNA markers linked to aphid resistance and a partial genetic linkage map were developed by bulk segregate analysis (BSA) and amplified fragment length polymorphism (AFLP) (Herselman et al., 2004). The F2:3 population derived from a cross with the aphid-resistant parent ICG 12991 were examined using a total of 308 AFLP primer combinations to find markers linked to aphid resistance. Twelve of the twenty putative markers were mapped to five linkage groups, spanned a map distance of 139.4 cM (Herselman et al., 2004). Recently, metabolomics with high performance liquid chromatography (HPLC) was employed to analyze the phenols fingerprinting of peanut plants with different resistance levels to aphid infestation, and common compounds such as the chlorogenic, syringic, quercetin, and ferulic acids were identified during aphid infestation (War et al., 2016). The quantities of the identified compounds are depending on genotypes and modes of aphids feeding.

#### 4.1.3 Helicoverpa armigera

In an effort to identify potential defense strategy, the biochemical basis of *H. armigera* infestation in peanut was analyzed with protein electrophoretic analysis and enzymatic assays (Harsulkar et al., 1999). Non-host peanut containing proteinase inhibitors (PIs) effectively inhibited the gut proteinases (HGPs) activity of *H. armigera* and larval growth (Harsulkar et al., 1999). Morphological traits were linked to resistance to *H. armigera* and can be used as markers of resistance selection. Significant correlations were found between main stem thickness, leaflet shape and length, hypanthium length, number of hairs on leaves, standard petal length and petal pattern, basal leaflet width, number of hairs, adherent length and width of stipules, plug length and *H. armigera* infestation (Sharma et al., 2003). Twelve resistance lines were selected under field and greenhouse conditions by screening 30 *Arachis* spp. (Sharma et al., 2003).

Salicylic acid (SA) and Jasmonic acid (JA) were also identified to induce defensive responses in peanut against *H. armigera* infestation. JA pretreatment markedly increases peroxidase (POD) and polyphenol oxidase (PPO) activities and high level total phenols, hydrogen peroxide (H_2_O_2_) and malondialdehyde (MDA) in peanut, and different levels of *H. armigera* resistance were recorded (War et al., 2011). Exogenous application of JA and SA induced resistance to *H. armigera*. Susceptible peanut and genotypes with different levels of *H. armigera* resistance showed higher amounts of secondary metabolites and levels enzymatic activities, and reduced *H. armigera* growth and development when pretreated with JA and SA in the green house (War et al., 2011). Recently, metabolomics with high performance liquid chromatography (HPLC) were employed to analyze the phenols fingerprinting of peanut plants with different resistance levels to *H. armigera* infestation (War et al., 2016). The observed common compounds were chlorogen, clove, quercetin and ferulic acid during *H. armigera* infestation (War et al., 2016).

#### 4.1.4 Spodoptera litura

Scientists have been trying to study the resistance of peanut to *S. litura* over the past few decades (Sharma et al., 2003). Some peanut morphological characteristics showed markedly correlation and/or regression coefficients with *S. litura* damage under field and greenhouse conditions, and were used as markers of selection for resistance to *S. litura* infection (Sharma et al., 2003). In addition, peanut grown under elevated carbon dioxide (CO_2_) level showed higher level carbon and polyphenols content, which reduced insect digestion efficiency, slowed down the growth of individual pest and reduced the *S. litura* pupation (Srinivasa et al., 2012). Furthermore, JA application can also boost the resistance to *S. litura* (War et al., 2011).

Genetic engineering has been proven to be effective in controlling insect pest. Ectopically expressing AhMPK3A, an *Arabidopsis* AtMPK3 gene homology in peanut, showed resistance to the first and second instar larvae of *S. litura* and generated higher expression levels of defense response genes such as PR1a, PR1b and LOX1 (Kumar et al., 2009). A chimeric Bt toxin protein cry1AcF with cry1Ac (domain I & II) and cry1F (domain III) was also employed to develop resistance peanut to *S. litura*. The cry1AcF transgenic peanut showed that cry1AcF significantly increased the mortality of *S. litura* larvae, and was effective against *S. litura* (Keshavareddy et al., 2013).

### 4.2 Peanut responses to microbial pathogens

#### 4.2.1 Fungi

##### 4.2.1.1 Leaf spot

For the past decades, many works have been done focusing on uncovering major gene for peanut leaf spot resistance, and masses of QTLs were identified (**Table 5**). The wild species accession, *A. cardenasii* GKP10017, is an important donor of leaf spot resistance to the peanut crop, because the segments from chromosome A02 and A03 correspond to some very strong QTLs that confer resistance to rust and LLS (Bertioli et al., 2021). Chu et al. (2019) discovered four leaf spot diseases resistance QTLs on both chromosome 3 and 5 in the Florida-07× GP-NC WS16 population. Zhang et al. (2020) identified two QTLs closely associating with resistance to ELS and LLS on chromosome B09 in the US mini-core peanut collection. In peanuts, many markers were recently found to be associated with QTLs for leaf spot disease resistance. Recent developments in mapping technologies for peanuts have identified of large numbers of QTL-associated polymorphic markers involved in peanuts ELS and LLS resistance. Eleven QTLs were found on a genetic map containing 56 microsatellites, or simple sequence repeat (SSR) marker loci through genetic mapping of GPBD4 (resistant varieties) derived a recombinant inbred line (RIL) population for LLS resistance (Khedikar et al., 2010), and 28 QTLs were identified on an improved genetic map with 260 SSR loci in the same RIL (Sujay et al., 2012). Two major QTLs for LLS resistance on chromosomes B10 and A03 were characterized based on mapping with 139 additional SSR and transposable element markers (Pandey et al., 2016). Nine QTLs involved in ELS resistance and 22 QTLs involved in LLS resistance were discovered and used in marker-assisted breeding (Pandey et al., 2017a). Khera et al. (2016) found 22 and 20 QTLs for ELS and LLS respectively, with a SSR-based map containing 248 loci in a population derived from SunOleic-97R × NC94022. QTLs for ELS and LLS were also recovered by utilizing single nucleotide polymorphism (SNP) linkage map, and identified a major QTL for late leaf spot resistance *via* analysis of interval sequences in peanut (Han et al., 2018). Zhou et al. (2016) used the Zhonghua 5 ICGV 86699 population for genetic mapping with 1685 SNPs from double-digested restriction-site associated DNA (ddRAD) sequences, and detected 20 LLS QTLs, among which 5 of the 6 major QTLs were located on chromosome B06. Liang et al. (2017) reported six QTLs on different chromosomes of ELS resistant parent Tx964117 were found using ddRAD-seq markers with 1211 loci developed from Tamrun OL07 × Tx964117 population. In a region harboring major QTLs for LLS and rust diseases, seven novel candidate expressed sequence tag-derived simple sequence repeat markers (EST-SSRs) related to stress were mapped using the F2 mapping population (GJG17 × GPBD4), and two major QTLs for LLS were found (Ahmad et al., 2020). In marker-assisted selection, the consensus QTLs across different genetic backgrounds are important and necessary. Shirasawa et al. (2018) pinpointed QTL candidates for LLS in a 1.4-Mb genome regions on A02, and selected four resistance-related genes as candidates for LLS in this region. Lu et al. (2018) identified one major Meta-QTL harboring 26 candidate genes for LLS in a region of about 0.38 cM. Moreover, QTL-seq approach was used to identify diagnostic markers and genomic regions for LLS resistance in peanut, and nine candidate genes with 17 SNPs were identified and one of these SNPs could serve as an allele-specific diagnostic marker (Pandey et al., 2017b). These delimited candidate genes-containing genomic regions will be valuable in uncovering the key resistant genes and in the development of LLS disease resistance in peanut breeding.

**Table 5:** QTL associated with Peanut responses to Leaf Spot.

	Location	Population	Reference
Leaf Spot	chromosomes B10 and A03	Zhonghua 5 x ICGV 86699	Zhang et al., 2017
	chromosome B06	Zhonghua 5 x ICGV 86699	Zhang et al., 2017
	chromosome 3 and 5	Florida-07× GP-NC WS16	Khera et al., 2016
	chromosome B06	Zhonghua 5 ICGV 86699	Zhou et al., 2016
	chromosome B09	GJG17 ×GPBD4	Zhang et al., 2017
	chromosome 3 and 5	Florida-07× GP-NC WS16	Chu et al. (2019)
	chromosome B09	US mini-core peanut collection	Zhang et al. (2020)
	A02 and A03	TAG 24× GPBD 4	Shirasawa et al., 2018
	chromosome A03	GJG17 × GPBD4	Ahmad et al., 2020
	on linkage group AhXV	TAG 24 x GPBD 4	Sujay et al., 2012
	A sub-genome	Tifrunner × GT-C20	Pandey et al., 2017a
	chromosomeA02, B04, B06, B09, and B10	Tamrun OL07 × Tx964117	Liang et al., 2017
	chromosomes A03, B04 and B05	Yuanza 9102	Han et al. (2017)

Transcriptome analysis was also performed in peanut leaf spot diseases. Cyclophilin gene with potential roles in peanut first line of defense are characterized using differential gene expression analysis following infection with the peanut late spot pathogen (Kumar and Kirti, 2011). Han et al. (2017) developed a highly susceptible M14 mutant to LLS derived from Yuanza 9102 cultivar. RNA-Seq analysis in M14 and the wild type Yuanza 9102 (resistant to several fungal diseases) leaf tissues under LLS pathogen challenge showed 2219 differentially expressed genes including 1317 up- and 902 down-regulated genes, including up-regulated pathogenesis-related (PR) protein genes, WRKY transcription factor genes, down-regulated chloroplast genes and depressed plant hormones in the M14 mutant. Furthermore, genes possibly involved in recognition events and early signaling responses to the pathogen, including resistance related proteins, hypersensitive cell death, cell wall strengthening and metabolism and signal transduction, were identified by transcriptomic and proteomic analyze (Kumar and Kirti, 2015). Dang et al. (2019) verified a group of 214 R-genes expressed in peanut leaves induced with leaf spot pathogen infection. Gong et al. (2020) identified 133 differentially expressed genes (DEGs) between ELS resistant and ELS susceptible peanut lines by transcriptome analysis, including leucine rich repeat (NLR) type resistance genes on the chromosome B2, peanut phytoalexin deficient 4 (PAD4) gene involved in NLR resistance proteins mediated immunity, and polyphenol oxidase (PPO) genes crucial to early leaf spot resistance in peanut. In a comparative transcriptome analysis from resistant line (GPBD 4), resistant introgression line (ICGV 13208) and a susceptible line (TAG 24), the resistance genes for LLS resistance, Aradu.P20JR and Aradu.Z87JB, were revealed on chromosome A02 and A03, respectively (Gangurde et al., 2021). Dang et al. (2021) reported 36 R-genes were markedly correlated with and differentially expressed in resistant lines. Most of the R-genes are receptor like kinases (RLKs) and receptor like proteins (RLPs) that function in precepting the presence of pathogen at the cell surface and initiating protection response.

In addition, metabolomics has also been applied to explore the mechanism of peanut response to LLS. LLS resistant peanut genotypes has higher levels secondary metabolites including but not limited to phenolic acid, flavanols, stilbenes and terpenoids (Mahatma et al., 2021).

##### 4.2.1.2 Rust

Mapping efforts have been concentrated on genomic sequence-based analysis and SNP enrichment mapping so as to define interested regions and identify candidate genes. Twelve QTLs for rust were identified by QTL analysis of 268 recombinant inbred lines from the rust segregation mapping population TAG 24 x GPBD 4. Interestingly a major QTL associated with rust resistance, as well as a candidate SSR marker (IPAHM 103) linked to this QTL, was identified and validated by both composite interval mapping and single-marker analysis using a broad range of resistant/susceptible breeding lines and progeny lines from another mapping population (TG 26 x GPBD 4) (Khedikar et al., 2010). Pasupuleti et al. (2016) used a marker-assisted backcross (MABC) method to import the major QTL accounting for 80% of the phenotypic variation (PV) in rust resistance. Nine candidate genes for rust resistance on chromosomes A03 spreading on a 3.06-Mb region were discovered with a QTL-Seq approach employed numerous resistance and susceptible lines from TAG 24 × GPBD 4 population (Pandey et al., 2017a). Five candidate genes for rust resistance within a 1.2 cM fragment on chromosomes A03 flanked by two SSR markers SSR_GO340445 and FRS 72 were also found with the help of mapping on the VG 9514 × TAG 24 population (Mondal and Badigannavar, 2018). A 2.7-Mb genome location of the rust resistance genes in the same genomic region of chromosome A03 was confirmed with the help of ddRAD-Seq and whole-genome resequencing for the population derived from hybrid between TAG 24 and GPBD 4 (Shirasawa et al., 2018). Recently, a major rust QTL containing resistance-related genes and R-genes functioning in inducing hypersensitive response (HR) during rust infection were validated by mapping with seven novel stress-related candidate EST-SSRs using 328 individuals from the F2 (GJG17×GPBD4) mapping population (Ahmad et al., 2020) (**Table 6**). In addition, nine rust resistant genotypes showed a 77% to 120% increase in pod yield under rust disease pressure over control, revealing significant environment (E) and genotype × environment (G×E) interactions (Chaudhari et al., 2019). QTL-Seq approach has been deployed to identify genomic regions and diagnostic markers for rust resistance in peanut, and 30 nonsynonymous SNPs affecting 25 candidate genes, and three allele-specific SNP diagnostic markers for rust resistance were identified (Pandey et al., 2017b). QTLs for rust resistance in the peanut wild species *A. magna* were developed using single-nucleotide polymorphism competitive allele-specific polymerase chain reaction markers and the marker function was validated in both diploid and tetraploid peanuts (Leal-Bertioli et al., 2015).

**Table 6:** QTL associated with Peanut responses to Rust.

	Location	Population	Reference
Rust	chromosomes A03	TAG 24 × GPBD 4	Pandey et al., 2017a
	chromosomes A03	VG 9514 × TAG 24	Mondal and Badigannavar, 2018
	chromosomes A03	TAG 24 and GPBD 4	Shirasawa et al., 2018
	chromosomes A03	GJG17×GPBD4	Ahmad et al., 2020
	chromosomes A06	TG 26 x GPBD 4	Khedikar et al., 2010

RNA-Seq data from susceptible peanut genotype JL-24 and resistant peanut genotype GPBD-4 revealed differentially expressed genes included pathogenesis-related (PR) proteins, ethylene-responsive factor, thaumatin, and F-box as well as R genes such as NBS-LRR upregulated in resistant genotype, whereas transcription factors such as WRKY, MYB, bZIP family down-regulated in susceptible genotype (Rathod et al., 2020). Using map-based cloning, a dominant rust resistance gene VG 9514-R located between FRS 72 and SSR_GO340445 markers in arahy03 chromosome was isolated and shown non-synonymous mutations in different protein domains (Mondal et al., 2022).

##### 4.2.1.3 Stem rot

By far, the QTLs and markers for have not discovered enough for peanut resistance to *S. rolfsii*. Utilizing QTL analysis with multi-season phenotyping and genotyping data from a TG37A×NRCG-CS85 population, 44 major epistatic QTLs explained phenotypic variation ranging from 14.32 to 67.95% were found (Dodia et al., 2019). Molecular mechanisms of peanuts resistant to *S. rolfsii* have been studied mostly with transcriptomic tools. The salicylic acid, defense-related signal molecule, peroxidase, marker enzymes, polymer lignin as well as the phenylalanine ammonia lyase-1,3-glucanase, all which related to the systemic acquired resistance and were observed could be induced by *S. rolfsii* derived elicitors (Nandini et al., 2010). RNA-Seq from infected peanut tissue found 12 differentially expressed genes including 7 genes related to defense in the plants and 3 genes related to virulence in the fungi (Jogi et al., 2016). Bosamia et al. (2020) unraveled genes encoding jasmonic acid pathway enzymes, receptor-like kinases, and transcription factors (TFs) including Zinc finger protein, WRKY, and C2-H2 zinc finger with high level expression in resistant peanut genotypes by RNA sequencing approaches. The pathogen-associated molecular patterns (PAMP)-triggered immunity was considered as a potential mechanism of stem rot resistance, while the jasmonic acid signaling pathway was deemed to a potential defense mechanism in peanut. There is also evidence of different enzymes activity in the crosstalk between peanut and *S. rolfsii* (Bosamia et al., 2020). *De-novo* genome sequencing of two distinct pathogen strains ZY and GP3 with high and weak aggressiveness respectively revealed the genomic basis for vary aggressiveness of *S. rolfsii* (Yan et al., 2021). The poll of pathogenicity-associated gene relate to aggressiveness were differed between GP3 and ZY based on comparative genomic analysis. GP3 and ZY possessed 58 and 45 unique pathogen-host interaction (PHI) genes, respectively. In addition, compared with GP3, ZY strain had more carbohydrateactive enzymes (CAZymes) in its secretome, especially the carbohydrate esterase (CBM), the polysaccharide lyase (PL) and the glycoside hydrolase (GH) family (Yan et al., 2021).

##### 4.2.1.4 Aspergillus flavus

Recently, series of QTLs in peanuts about resistance to *A. flavus* infection were successfully identified by QTL mapping (**Table 7**). Yu et al. (2019) identified two QTLs with 7.96 and 12.16% phenotypic variation explained (PVE) on chromosomes A03 and A10, respectively by constructing a genetic map with 1,219 SSR loci and a recombinant inbred line (RIL) population resulted from crossing ICG12625 with susceptible cultivar Zhonghua 10. Khan et al. (2020) identified a major QTL with a PVE of 18.11% on A03 and a minor QTL with a PVE of 4.4% on B04 by utilizing SNP based genetic map using specific length amplified fragment sequencing (SLAF-Seq). Based on 200 recombinant inbred lines (RILs) mapping population from a hybrid of a susceptible variety Zhonghua 16 with resistant germplasm J11; Jiang et al. (2021) reported six novel resistant QTLs on chromosomes A05, A08, B01, B03, and B10 with 5.03–10.87% PVE, respectively.

**Table 7:** QTL associated with Peanut responses to *Aspergillus flavus.*.

	Location	Population	Reference
*Aspergillus* *flavus*	chromosomes A03 and A10	ICG12625× Zhonghua 10	Yu et al., 2019
	chromosomes A03 and B04	Zhonghua 16 × J11	Khan et al., 2020
	chromosomes A05, A08, B01, B03, and B10	Zhonghua 16 × J11	Jiang et al., 2021

The molecular mechanisms of peanut–*A. flavus* interactions and peanut resistance to aflatoxin generation need to be studied to create effective countermeasures against postharvest aflatoxin contamination. A great number of transcriptome analysis gave hints and information to this aspect. Guo et al. (2008) constructed cDNA libraries from the seeds of peanut resistant cultivars (GT-C20) and susceptible cultivars (Tifrunner) with 21777 EST sequences. Using two-dimensional electrophoresis, mass spectrometry and real-time RT-PCR; Wang et al. (2010) reported the identification of twelve potentially differentially expressed proteins between resistant peanut variety YJ-1 and susceptible peanut variety Yueyou 7 under conditions of sufficient water, drought stress and flavus infection with drought stress. According to a peanut oligonucleotide microarray chip analysis, 62 genes with upregulated expression in resistant cultivar and 22 putative Aspergillus-resistance genes with high level expression in the resistant cultivar were determined (Guo et al., 2011). In addition, a series of aflatoxins-responsive proteins, including those involved in immune signaling and innate immunity, induction of defense, PAMP perception, penetration resistance, hypersensitive response, DNA and RNA stabilization, biosynthesis of phytoalexins, condensed tannin synthesis, cell wall responses, peptidoglycan assembly, detoxification and metabolic regulation, were identified in peanut cotyledons infected with aflatoxin-producing (toxigenic) but not non-aflatoxinogenic (toxigenic) *A. flavus* strains, using a differential proteomics approach (Wang et al., 2012). Global transcriptome analysis of post-harvest peanut seeds of susceptible (Zhonghua 12) and resistant (Zhonghua 6) peanut genotypes undergoing fungal infection and aflatoxin production revealed 128, 725 unigenes, of which 30, 143 were differentially expressed, and 842 are potential defense-related genes, including pathogenesis-related proteins, leucine-rich repeat receptor-like kinases, transcription factors, mitogen-activated protein kinase, nucleotide binding site-leucine-rich repeat proteins, polygalacturonase inhibitor proteins, ADP-ribosylation factors and other defense-related crucial factors (Wang et al., 2016). Transcriptomic and proteomic analyses identified 663 DEGs and 314 differentially expressed proteins during the infection of J11 peanut by *A. flavus* (Zhao et al., 2019). Transcriptomic network analysis from the publically available RNA-seq datasets of resistant and susceptible peanut cultivars infected with *A. flavus* revealed a series of candidate genes involved in resistance response against *A. flavus*, including genes encoding R proteins, pattern recognition receptor genes, protein P21, laccase, thaumatin-like protein 1b and pectinesterases (Jayaprakash et al., 2021). Core genes positively associated with peanut resistance to *A. flavus* were determined by weighted gene coexpression network analysis (WGCNA) and comparative transcriptome approach (Cui et al., 2022). About 18 genes encoding pattern recognition receptors (PRRs), MAPK kinase, serine/threonine kinase (STK), 1 aminocyclopropane-1-carboxylate oxidase (ACO1), pathogenesis related proteins (PR10), phosphatidylinositol transfer protein, SNARE protein SYP121, cytochrome P450, pentatricopeptide repeat (PPR) protein and pectinesterase, might contribute to peanut resistance to *A. flavus* (Cui et al., 2022).

Metabolite and miRNA profiling work for *A. flavus* resistance were also reported in peanut. Sharma et al. (2021) found that the pipecolic acid (Pip) was a key component of peanut resistance to *A. flavus* by performing untargeted metabolite profiling. And the function of Pip against *A. flavus* was validated by employing multiple resistant and susceptible peanut cultivars. Correlation analysis of small RNAs, transcriptomes and degradomes revealed a total number of 447 genes, 30 miRNAs and 21 potential miRNA/mRNA pairs showing significantly differential expression when resistant cultivar (GT-C20) and susceptible cultivar (Tifrunner) were treated with *A. flavus*. The accumulation of flavonoids in resistant and susceptible genotypes might be regulated by miR156/SPL pairs and the NBS-LRR gene expression level in resistant genotype might be regulated by miR482/2118 family (Zhao et al., 2020).

#### 4.2.2 Viruses

TSWV, a single-stranded RNA virus, is one of the most pathogenic viruses in peanut. TSWV not only caused spotted wilt disease but also constrain peanut yield (Garcia et al., 2000). To better understand the mechanisms of peanut-TSWV interactions and peanut resistance to TSWV, QTL mapping was used to locate QTL for TSWV resistance (**Table 8**). The first genetic linkage map based on NC94022-derived population was constructed and a substantial QTL with a PVE of 35.8% associated with resistance to TSWV on linkage group A01 was identified (Qin et al., 2012). An enhanced genetic map from the same population was constructed and the QTLs related to multi-year TSWV phenotypic data on chromosome A01 were identified (Khera et al., 2016). About 48 QTLs with phenotypic variance explained (PVE) ranging from 3.88 to 29.14% and six QTLs associated with spotted wilt resistance were identified, among which five QTLs were found on the A01 and the other one located on A09 chromosome (Khera et al., 2016). A major QTL on chromosome A01 flanked by marker AHGS4584 and GM672, associated with spotted wilt disease with up to 22.7% PVE in a spotted wilt resistant cultivar Florida-EP™ 113 was identified based on phenotypic data, and 2,431 SSR markers were screened from the two parental lines whole peanut genome (Tseng et al., 2016). Three QTLs on chromosome A01 of RIL derived from peanut lines of SunOleic 97R and NC94022 were identified using the whole genome re-sequencing approach, among which one QTL had the greatest impact on phenotypic variation, reaching 36.51%, including one 89.5 Kb genomic region with a set of genes coding for NBS-LRR proteins, strictosidine synthase-like, and chitinases (Agarwal et al., 2019). Recently, 11 QTLs for TSWV resistance were discovered using the recombinant inbred line (RIL) mapping population of “Tifrunner × GT-C20” (Pandey et al., 2017a). Zhao et al. (2018) refined the resistance QTL to a 0.8 Mb region on A01 chromosome with SSR markers.

**Table 8:** QTL associated with Peanut responses to TSWV.

	Location	Population	Reference
tomato spotted wilt virus(TSWV)	chromosome A01	NC94022	Qin et al., 2012
	chromosome A01	NC94022	Khera et al., 2016
	chromosomeA09 and A01	SunOleic 97R × NC94022	Khera et al., 2016
	chromosome A01	Florida-EP™	Tseng et al., 2016
	chromosome A01	SunOleic 97R×NC94022	Agarwal et al., 2019
	chromosome A01	Tifrunner × GT-C20	Pandey et al., 2017a

PStV and other viruses may also infect peanut (Singh et al., 2009). However, studies on the mechanism of peanut response to various viruses using Omics technology are very few. Some reports indicate that transgenic peanuts carrying viral gene fragments can improve their antiviral ability. For example, peanut lines carrying viral coat protein gene sequences, exhibited high resistance levels to PStV (Higgins et al., 2004). Genetic engineering of peanut using genes encoding the nucleocapsid protein (N gene) of peanut bud necrosis virus was also tested against bud necrosis disease in peanut, a disease for which no persistent resistance in the existing germplasm (Rao et al., 2013). Recent research found that PeaeIF4E and PeaeIF(iso)4E, the eukaryotic translation initiation factors played important roles in PStV infection, and the silencing of PeaeIF4E and PeaeIF(iso)4E genes significantly weakened PStV accumulation in peanut (Xu et al., 2017).

#### 4.2.3 Bacterial

*R. solanacearum* has a wide host range and strong long-term survival ability, making it very difficult to eradicate. In the past decade, progress has been made in the genetic behavior, trait mapping, gene discovery and diagnostic markers of peanut bacterial wilt resistance. Based on SSR and AFLP analyses, the genetic relationships of 31 peanut genotypes with different resistance levels to *R. solanacearum* were assessed, and four SSR primers and one AFLP primer were found to be effective (Jiang et al., 2007). Linkage map analysis using SSR and SNP markers found two major QTLs associated with *R. solanacearum* resistance on linkage groups LG01 and LG10 with PVE of 12 to 21% (Zhao et al., 2016). A major and stable QTL for bacterial wilt resistance (qBWB02.1) on chromosome B02 was validated by a high-density SNP map with a RIL population from the hybrid Yuanza YZ9012 with Xuzhou68-4 (Wang et al., 2018). Meanwhile, a QTL in the same linkage region was confirmed by BSA based on sequencing-based trait mapping approach and QTL-seq for recombinant inbred line (RIL) derived from a cross between cultivars Yuanza 9102 and Xuzhou 68-4 (Luo et al., 2019). In addition, two major QTLs on chromosome B02 were verified through linkage mapping and QTL-seq on the basic of a RIL population produced from the hybrid between peanut cultivars Zhonghua6 and Xuhua13 and were further validated by two diagnostic markers (Luo et al., 2020). This same major QTL on chromosome B02 was repeatedly identified with different methods and RIL populations (Wang et al., 2018; Luo et al., 2020), suggesting that it is the main QTL for peanut resistance to bacterial wilt. By applying KASP markers that were polymorphic between the two parents, the major QTL qBWA12, for resistance of peanut against bacterial, was fine mapped to a 216.7 kb region based on whole-genome resequencing data (Qi et al., 2022) (**Table 9**).

**Table 9:** QTL associated with Peanut responses to Bacterial wilt.

	Location	Population	Reference
Bacterial wilt	chromosome B02	Yuanza YZ9012 × Xuzhou68-4	Wang et al., 2018
	chromosome B02	Zhonghua6 and Xuhua13	Luo et al., 2020
	mapped to a 216.68 kb physical region	*A. hypogaea*	Qi et al., 2022

Beside the QTLs and markers, a number of peanut genes associated with *R. solanacearum* interactions were also reported. Using complementary DNA amplified length polymorphism (cDNA-AFLP) technique; Peng et al. (2011) studied a BW-sensitive cultivar, ‘Zhonghua 12’, and a BW-resistant one, ‘Yuanza 9102’ upon *R. solanacearum* infection, analyzed differential expression of genes associated to BW-resistance, and found 40 transcript-derived fragments (TDFs) closely related to BW resistance, which encode proteins associated with cell structure or/and protein synthesis, defense, energy, signal transduction, metabolism, cell growth and transcription,. Huang et al. (2012) screened differentially expressed genes, including those involved in jasmonic acid and ethylene signal transduction, from peanut cDNA libraries of *R. solanacearum* challenged roots and leaves. Chen et al. (2014) performed global transcriptome profiling on the *R. solanacearum*-infected roots of peanut susceptible (S) and resistant (R) genotypes, and found that the down-regulation of primary metabolism and the genotype-specific expression pattern of defense related DEGs (R gene, cell wall genes, LRR-RLK, etc.) contributing to the resistance difference between S and R genotypes. Recently, 174 WRKY genes (AhWRKY) were identified from the cultivated peanut genome, their differential expression patterns were analyzed in sensitive and resistant peanut genotypes infected with the *R. solanacearum*, and the possible roles of candidate WRKY genes were identified in peanut resistance against *R. solanacearum* infection (Yan et al., 2022). A series of candidate genes with possible bacterial wilt resistance were directly cloned from peanut and functionally studied. Overexpression of the peanut AhRRS5 (a novel peanut NBS-LRR gene) or the AhRLK1 (CLAVATA1-like leucine-rich repeat receptor-like kinase) or the AhGLK1b (GOLDEN2-like Transcription Factor) enhanced plant disease resistance to *R. solanacearum* (Zhang et al., 2017; Zhang et al., 2019; Ali et al., 2020).

## 5 Application of omics research in breeding program

In the last two decades, using the most successful approach namely marker-assisted selection (MAS) or marker-assisted backcrossing (MABC), diagnostic markers have successfully been developed in groundnut for resistance to nematode, rust and LLS. The first excellent example is the marker-assisted improvement of popular cultivar for nematode resistance in USA. The discovery of resistance to *M. arenaria* in wild *Arachis* species and the interspecific hybrid, TxAG-6, allowed the development of the first peanut cultivar resistant to *M. arenaria* (Simpson and Starr, 2001). The interspecific *Arachis* hybrid TxAG-6 was the source of this resistance and the donor parent in a backcross breeding program to introgress resistance into cultivated peanut. Gene segments with resistance from TxAG-6 were introduced into peanut cultivars through an interspecific hybridization backcrossing, two root-knot nematode-resistant varieties, COAN and NemaTAM were first developed with marker-assisted backcrossing is in the USA (Simpson and Starr, 2001; Simpson et al., 2003; Church et al., 2005). By far, at least other four commercial nematode-resistant cultivars (Tifguard, Webb, TifN/V OL, and Georgia 14N) resulting from this cross have been released (Denwar et al., 2021).

The major peanut rust resistance-related QTLs and markers were almost mapped from two recombinant inbred line (RIL) mapping populations, namely ‘TAG 24’ (susceptible) × ‘GPBD 4’ (resistant) and ‘TG 26’ (susceptible) × ‘GPBD 4’ (Khedikar et al., 2010; Sujay et al., 2012). The QTL region controlling rust resistance, including the disease resistance-linked markers (IPAHM103, GM2079, GM1536 and GM2301) from the disease-resistant donor GPBD 4, were introgressed into two elite peanut varieties (‘TAG 24’ and ‘ICGV 91114’) and one old but popular variety (‘JL 24’) through MABC in India. The backcrossed homozygous lines (BC3F2) were obtained in just three years of time and shown significant increase in rust resistance (Varshney et al., 2014). In addition, TMV2 is a very popular groundnut variety among the Indian farmers but is highly susceptible to LLS and rust. The LLS and rust resistance linked markers (GM2009, GM2079, GM2301, GM1839 and IPAHM103) from the disease-resistant donor GPBD 4 were introgressed into TMV2 using MABC approach, and two homozygous backcross lines, namely TMG‐29 and TMG‐46, were obtained which showed resistance to rust and LLS (Kolekar et al., 2017). Recently, the above linked and validated markers for resistance to rust and LLS were used to improve three popular Indian cultivars (GJG 9, GG 20, and GJGHPS 1) using MABC, and the Phenotyping of the ILs, using the 58 K SNP array for assessing background genome recovery across the chromosomes (Pandey et al., 2017c), revealed their disease resistance scores comparable to the resistant parent GPBD 4 (Shasidhar et al., 2020).

## 6 Conclusion and perspective

This review summarizes various omics studies in peanut biotic stress in the past two decades emphasizing on peanut resistance to various biotic pests and pathogens. Progresses in the field of peanut responding to biotic stress are accumulating, and large amount of omics data have been produced to decipher the molecule clues between peanut and the infesting agents. In the implementation of marker-assisted selection, the first step is the identification of genomic regions conferring disease resistance in wild species, which can speed up the introgression of wild disease resistance genes with less linkage drag. Also, the identified disease resistance genes can be deployed in biotechnology and genomics-assisted breeding for the development of disease resistant cultivars to reduce the yield loss in peanut production. However, the accumulated data over the past years are intertwined and disorganized, and have not been rationally sorted out. The technology that produces large amount of Omics data has surpassed our ability to analyze and utilize them. Big data is a common phenomenon of this era, but it is extremely urgent to develop technology in dealing with the Omics data, and most importantly to use the Omics data in crop breeding for better resistance to biotic and abiotic stresses.

Due to the peanut genome complexity, research on peanut Omics are relatively lagged as compared with other crops as well as with the model plant species. Not many results are obtained from peanut biotic stresses by using the Omics tools such as transcriptomics, proteomics and other technologies. For the major peanut pests and pathogens, efforts are mostly on the most important pests and diseases such as the root-knot nematodes, insects and fungi, while less attention is paid to the viral and bacterial pathogens. Thus, more efforts are needed in future Omics research on biotic stresses affecting peanuts.

In addition, results from Omics data including putative QTLs, molecular markers, candidate genes, metabolites, proteins, etc., need to be cross tested with wet lab research before they can be reliably used in scientific research and commercial breeding. For those coarsely mapped QTLs, other technologies may be needed to precisely map them to a smaller region on the corresponding chromosome, and eventually clone them. Candidate genes that may play a role in peanut disease and insect resistance, functional genetics are needed to overexpress them in transgenic research or their mutations need to be created by CRISPR genome editing technology to verify their related functions. On the other hand, Omics data are valuable in genome editing to design efficient CRISPR targets, with reduced off-target mutations in crop breeding. With the success of peanut genome editing with the CRISPR technology, it is possible to use Omics data in peanut molecular breeding for pest and disease resistance (Zhang et al., 2021).

## Author contributions

RH, SZ, HL, CG and WY write this manuscript. All authors contributed to the article and approved the submitted version.

## References

[B1] Agarwal G. Clevenger J. Kale S. M. Wang H. Pandey M. K. Choudhary D.. (2019). A recombination bin-map identified a major QTL for resistance to tomato spotted wilt virus in peanut (*Arachis hypogaea*). Sci. Rep. 9, 18246. doi: 10.1038/s41598-019-54747-1 31796847 PMC6890646

[B2] Agarwal G. Clevenger J. Pandey M. K. Wang H. Shasidhar Y. Chu Y.. (2018). High-density genetic map using whole-genome resequencing for fine mapping and candidate gene discovery for disease resistance in peanut. Plant Biotechnol. J. 16 (11), 1954–1967. doi: 10.1111/pbi.12930 29637729 PMC6181220

[B3] Agrios G. N. (2005). “Plant diseases caused by nematodes,” in Introduction to plant pathology, ffth ed (London: Elsevier Academic Press), 826–872.

[B4] Ahmad S. Nawade B. Sangh C. Mishra G. P. Bosamia T. C. Kumar. N.. (2020). Identification of novel QTLs for late leaf spot resistance and validation of a major rust QTL in peanut (*Arachis hypogaea l.*). 3 Biotech. 10, 458. doi: 10.1007/s13205-020-02446-4. T. R. PMC7527388 33088655

[B5] Ali N. Chen H. Zhang C. Khan S. A. Gandeka M. Xie D.. (2020). Ectopic expression of AhGLK1b (GOLDEN2-like transcription factor) in arabidopsis confers dual resistance to fungal and bacterial pathogens. Genes (Basel) 11, 343. doi: 10.3390/genes11030343 32213970 PMC7141132

[B6] Anco D. J. Thomas J. S. Jordan D. L. Shew B. B. Monfort W. S. Mehl H. L.. (2020). Peanut yield loss in the presence of defoliation caused by late or early leaf spot. Plant Dis. 104 (5), 1390–1399. doi: 10.1094/PDIS-11-19-2286-RE 32223639

[B7] Arya S. S. Salve A. R. Chauhan S. (2015). Peanuts as functional food: a review. J. Food Sci. Technol. 53 (1), 31–41. doi: 10.1007/s13197-015-2007-9 26787930 PMC4711439

[B8] Ballén-Taborda C. Chu Y. Ozias-Akins P. Holbrook C. C. Timper P. Jackson S. A.. (2022). Development and genetic characterization of peanut advanced backcross lines that incorporate root-knot nematode resistance from *Arachis* stenosperma. Front. Plant Sci. 12. doi: 10.3389/fpls.2021.785358 PMC8801422 35111175

[B9] Ballén-Taborda C. Chu Y. Ozias-Akins P. Timper P. Holbrook C. C. Jackson S. A.. (2019). A new source of root-knot nematode resistance from *Arachis* stenosperma incorporated into allotetraploid peanut (*Arachis hypogaea*). Sci. Rep. 9, 17702. doi: 10.1038/s41598-019-54183-1 31776412 PMC6881346

[B10] Ballén-Taborda C. Chu Y. Ozias-Akins P. Timper P. Jackson S. Bertioli D.. (2021). Validation of resistance to root-knot nematode incorporated in peanut from the wild relative *Arachis stenosperma* . Agron. J. 113, 2293–2230. doi: 10.1002/agj2.20654

[B11] Bertioli D. J. Cannon S. B. Froenicke L. Huang G. Farmer A. D. Cannon E. K.. (2016). The genome sequences of *Arachis duranensis* and *Arachis ipaensis*, the diploid ancestors of cultivated peanut. Nat. Genet. 48, 438–446. doi: 10.1038/ng.3517 26901068

[B12] Bertioli D. J. Clevenger J. Godoy I. J. Stalker H. T. Wood S. Santos J. F.. (2021). Legacy genetics of *Arachis* cardenasii in the peanut crop shows the profound benefits of international seed exchange. Proc. Natl. Acad. Sci. U.S.A. 118 (38), e2104899118. doi: 10.1073/pnas.2104899118 34518223 PMC8463892

[B13] Bertioli D. J. Jenkins J. Clevenger J. Dudchenko O. Gao D. Seijo G.. (2019). The genome sequence of segmental allotetraploid peanut *Arachis* hypogaea. Nat. Genet. 51, 877–884. doi: 10.1038/s41588-019-0405-z 31043755

[B14] Bertioli D. J. Leal-Bertioli S. C. Lion M. B. Santos V. L. Pappas G. Cannon S. B.. (2003). A large scale analysis of resistance gene homologues in *Arachis* . Mol. Genet. Genomics 270, 34–45. doi: 10.1007/s00438-003-0893-4 12928866

[B15] Bi Y. P. Li G. C. Wang X. L. Li J. W. Shan L. Guo B. T.. (1999). Cloning and sequencing of peanut stripe virus coat protein gene. J. Agric. Biotechnol. 7, 211–214.

[B16] Blackman R. L. Eastop V. F. (2007). “Taxonomic issues,” in Aphids as crop pests. Eds. van Emden H. F. Harrington R. (U. K: CAB International), 1–29.

[B17] Blount A. R. Pittman R. N. Smith B. A. Morgan R. N. Dankers W. Sprenkel R. K.. (2002). First report of peanut stunt virus in perennial peanut in north Florida and southern Georgia. Plant Dis. 86, 326. doi: 10.1094/PDIS.2002.86.3.326C 30818617

[B18] Bosamia T. C. Dodia S. M. Mishra G. P. Ahmad S. Joshi B. Thirumalaisamy P. P.. (2020). Unraveling the mechanisms of resistance to sclerotium rolfsii in peanut (*Arachis hypogaea l.*) using comparative RNA-seq analysis of resistant and susceptible genotypes. PloS One 15, e0236823. doi: 10.1371/journal.pone.0236823 32745143 PMC7398544

[B19] Chaudhari S. Khare D. Patil S. C. Sundravadana S. Variath M. T. Sudini H. K.. (2019). Genotype × environment studies on resistance to late leaf spot and rust in genomic selection training population of peanut (*Arachis hypogaea l.*). Front. Plant Sci. 10. doi: 10.3389/fpls.2019.01338 PMC6904303 31867023

[B20] Chen K. R. Xu Z. Y. Zhang Z. Y. Chen J. X. (1990). Seed transmission of peanut stripe virus (PStV) in peanut III. the changes of the rate of PStV infected peanut seeds between high and low seed-borne lines at the peanut pods development. Chin. J. Oil Crop Sci. 4, 79–82.

[B21] Chen Y. Ren X. Zhou X. Huang L. Yan L. Lei Y.. (2014). Dynamics in the resistant and susceptible peanut (Arachis hypogaea l.) root transcriptome on infection with the ralstonia solanacearum. BMC Genomics 15. doi: 10.1186/1471-2164-15-1078 PMC4300042 25481772

[B22] Choi H. S. Kim J. S. Cheon J. U. Choi J. K. Pappu S. S. Pappu H. R. (2001). First report of peanut stripe virus (Family potyviridae) in south Korea. Plant Dis. 85, 679. doi: 10.5423/PPJ.2006.22.1.097 30823042

[B23] Choi H. S. Kim M. Park J. W. Cheon J. U. Kim K. H. Kim J. S.. (2006). Occurrence of bean common mosaic virus (BCMV) infecting peanut in Korea. Plant Pathol. J. 22, 97–102. doi: 10.5423/PPJ.2006.22.1.097

[B24] Chu Y. Chee P. Culbreath A. Isleib T. G. Holbrook C. C. Ozias-Akins P. (2019). Major QTLs for resistance to early and late leaf spot diseases are identified on chromosomes 3 and 5 in peanut (*Arachis hypogaea*). Front. Plant Sci. 10. doi: 10.3389/fpls.2019.00883 PMC6625158 31333711

[B25] Church G. T. Starr J. L. Simpson C. E. (2005). A recessive gene for resistance to meloidogyne arenaria in interspecific *Arachis* spp. hybrids. J. Nematol 37 (2), 178–184. 19262858 PMC2620960

[B26] Clevenger J. Chu Y. Chavarro C. Agarwal G. Bertioli D. J. Leal-Bertioli S. C. M.. (2017). Genome-wide SNP genotyping resolves signatures of selection and tetrasomic recombination in peanut. Mol. Plant 10 (2), 309–322. doi: 10.1016/j.molp.2016.11.015 27993622 PMC5315502

[B27] Cui M. Han S. Wang D. Haider M. S. Guo J. Zhao Q.. (2022). Gene Co-expression network analysis of the comparative transcriptome identifies hub genes associated with resistance to aspergillus flavus l. @ in cultivated peanut (*Arachis hypogaea l.*). Front. Plant Sci. 13. doi: 10.3389/fpls.2022.899177 PMC9264616 35812950

[B28] Culbreath A. K. Srinivasan R. (2011). Epidemiology of spotted wilt disease of peanut caused by tomato spotted wilt virus in the southeastern u. s. Virus Res. 159, 101–109. doi: 10.1016/j.virusres.2011.04.014 21620508

[B29] Culbreath A. K. Todd J. W. Brown S. L. (2003). Epidemiology and management of tomato spotted wilt in peanut. Annu. Rev. Phytopathol. 41, 53–75. doi: 10.1016/j.virusres.2009.01.009 12704217

[B30] Dang J. L. Horvath D. M. Staskawicz B. J. (2013). Pivoting the plant immune system from dissection to deployment. Science 341, 746–751. doi: 10.1126/science.1236011 23950531 PMC3869199

[B31] Dang P. M. Lamb M. C. Bowen K. L. Chen C. Y. (2019). Identifcation of expressed r-genes associated with leaf spot diseases in cultivated peanut. Mol. Biol. Rep. 46, 225–239. doi: 10.1007/s11033-018-4464-5 30498882

[B32] Dang P. M. Lamb M. C. Chen C. Y. (2021). Association of differentially expressed r-gene candidates with leaf spot resistance in peanut (*Arachis hypogaea l.*). Mol. Biol. Rep. 48, 323–334. doi: 10.1007/s11033-020-06049-3 33403558 PMC7884587

[B33] Decraemer W. Hunt D. (2006). “Structure and classifcation,” In Perry R. N. Moens M. (Eds.), Plant Nematology, pp. 3–32.

[B34] Demski J. W. Lovell G. R. (1985). Peanut stripe virus and the distribution of peanut seed. Plant Dis. 69, 734–738.

[B35] Denwar N. N. Simpson C. S. Starr J. L. Wheeler T. A. Burow M. D. (2021). Evaluation and selection of interspecific lines of groundnut (*Arachis hypogaea* l.) for resistance to leaf spot disease and for yield improvement. Plants (Basel) 10 (5), 873. doi: 10.3390/plants10050873 33926071 PMC8146533

[B36] Dodia S. M. Joshi B. Gangurde S. S. Thirumalaisamy P. P. Mishra G. P. Narandrakumar D.. (2019). Genotyping-by-sequencing based genetic mapping reveals large number of epistatic interactions for stem rot resistance in groundnut. Theor. Appl. Genet. 132, 1001–1016. doi: 10.1007/s00122-018-3255-7 30539317

[B37] Dong W. B. Holbrook C. C. Timper P. Brenneman T. B. Chu Y. Ozias-Akins P. (2008). Resistance in peanut cultivars and breeding lines to three root-knot nematode species. Plant Dis. 92, 631–638. doi: 10.1094/PDIS-92-4-0631 30769645

[B38] Gangurde S. S. Nayak S. N. Joshi P. Purohit S. Sudini H. K. Chitikineni A.. (2021). Comparative transcriptome analysis identified candidate genes for late leaf spot resistance and cause of defoliation in groundnut. Int. J. Mol. Sci. 22, 4491. doi: 10.3390/ijms22094491 33925801 PMC8123497

[B39] Garcia L. E. Brandenburg R. L. Bailey J. E. (2000). Incidence of tomato spotted wilt virus (Bunyaviridae) and tobacco thrips in virginiatype peanuts in north Carolina. Plant Dis. 84, 459–464. doi: 10.1094/PDIS.2000.84.4.459 30841170

[B40] Garcia G. M. Stalker H. T. Kochert G. (1995). Introgression analysis of an interspecific hybrid population in peanuts (*Arachis hypogaea l.*) using RFLP and RAPD markers. Genome 38, 166–176. doi: 10.1139/g95-021 7729680

[B41] Genin S. (2010). Molecular traits controlling host range and adaptation to plants in ralstonia solanacearum. New Phytol. 187, 920–928. doi: 10.1111/j.1469-8137.2010.03397.x 20673287

[B42] Gong L. Han S. Yuan M. Ma X. Hagan A. He G. (2020). Transcriptomic analyses reveal the expression and regulation of genes associated with resistance to early leaf spot in peanut. BMC Res. Notes 13, 381. doi: 10.1186/s13104-020-05225-9 32782019 PMC7418390

[B43] Grichar W. J. Besler B. A. Jaks A. J. (1998). Groundnut (Arachis hypogaea l.) cultivar response to leaf spot disease development under four disease management programs. Peanut Sci. 25, 35–39. doi: 10.3146/i0095-3679-25-1-9

[B44] Guimaraes P. M. Guimaraes L. A. Morgante C. V. Silva O. Araujo A. C. Martins A. C.. (2015). Root transcriptome analysis of wild peanut reveals candidate genes for nematode resistance. PloS One 10, e0140937. doi: 10.1371/journal.pone.0140937 26488731 PMC4619257

[B45] Guo B. Chen Z.-Y. Lee R. D. Scully B. T. (2008). Drought stress and preharvest aflatoxincontamination in agricultural commodity: genetics, genomics and proteomics. J. Integr. Plant Biol. 50, 1281–1291. doi: 10.1111/j.1744-7909.2008.00739.x 19017115

[B46] Guo B. Fedorova N. D. Chen X. Wan C. H. Wang W. Nierman W. C.. (2011). Gene expression profiling and identification of resistance genes to aspergillus flavus infection in peanut through EST and microarray strategies. Toxins (Basel) 3, 737–753. doi: 10.3390/toxins3070737 22069737 PMC3202856

[B47] Haas R. Zelezniak A. Iacovacci J. Kamrad S. Townsend S. Ralser M.. (2017). Designing and interpreting 'multi-omic' experiments that may change our understanding of biology. Curr. Opin. Syst. Biol. 6, 37–45. doi: 10.1016/j.coisb.2017.08.009 32923746 PMC7477987

[B48] Han S. Liu H. Yan M. Qi F. Wang Y. Sun Z.. (2017). Differential gene expression in leaf tissues between mutant and wild-type genotypes response to late leaf spot in peanut (*Arachis hypogaea l.*). PloS One 12, e0183428. doi: 10.1371/journal.pone.0183428 28841668 PMC5571927

[B49] Han S. Yuan M. Clevenger J. P. Li C. Hagan A. Zhang X.. (2018). A SNP-based linkage map revealed QTLs for resistance to early and late leaf spot diseases in peanut (*Arachis hypogaea l.*). Front. Plant Sci. 9. doi: 10.3389/fpls.2018.01012 PMC6048419 30042783

[B50] Harsulkar A. M. Giri A. P. Patankar A. G. Gupta V. S. Sainani M. N. Ranjekar P. K. (1999). Deshpande VV. successive use of non-host plant proteinase inhibitors required for effective inhibition of helicoverpa armigera gut proteinases and larval growth. Plant Physiol. 121, 497–506. doi: 10.1104/pp.121.2.497 10517841 PMC59412

[B51] Hayward A. C. (1991). Biology and epidemiology of bacterial wilt caused by pseudomonas solanacearum. Annu. Rev. Phytopathol. 29, 65–87. doi: 10.1146/annurev.py.29.090191.000433 18479193

[B52] Herselman L. Thwaites R. Kimmins F. M. Courtois B. van der, Merwe. P. J. Seal S. E. (2004). Identification and mapping of AFLP markers linked to peanut (*Arachis hypogaea l.*) resistance to the aphid vector of groundnut rosette disease. Theor. Appl. Genet. 109, 1426–1433. doi: 10.1007/s00122-004-1756-z 15290049

[B53] Higgins C. M. Hall R. M. Mitter N. Cruickshank A. Dietzgen R. G. (2004). Peanut stripe potyvirus resistance in peanut (*Arachis hypogaea l.*) plants carrying viral coat protein gene sequences. Transgenic Res. 13, 59–67. doi: 10.1023/b:trag.0000017166.29458.74 15070076

[B54] Holbrook C. Stalker H. (2003). Peanut breeding and genetic resources. Plant Breed Rev. 22, 297–356. doi: 10.1002/9780470650202.ch6

[B55] Huang J. Yan L. Lei Y. Jiang H. Ren X. Liao B. (2012). Expressed sequence tags in cultivated peanut (*Arachis hypogaea*): discovery of genes in seed development and response to ralstonia solanacearum challenge. J. Plant Res. 125, 755–769. doi: 10.1007/s10265-012-0491-9 22648474

[B56] Jayaprakash A. Roy A. Thanmalagan R. R. Arunachalam A. Ptv L. (2021). Immune response gene coexpression network analysis of *Arachis hypogaea* infected with aspergillus flavus. Genomics 113, 2977–2988. doi: 10.1016/j.ygeno.2021.06.027 34153499

[B57] Jiang H. Liao B. Ren X. Lei Y. Mace E. Fu T.. (2007). Comparative assessment of genetic diversity of peanut (*Arachis hypogaea l.*) genotypes with various levels of resistance to bacterial wilt through SSR and AFLP analyses. J. Genet. Genomics 34, 544–554. doi: 10.1016/S1673-8527(07)60060-5 17601614

[B58] Jiang Y. Luo H. Yu B. Ding Y. Kang Y. Huang L.. (2021). High-density genetic linkage map construction using whole-genome resequencing for mapping QTLs of resistance to aspergillus flavus infection in peanut. Front. Plant Sci. 12. doi: 10.3389/fpls.2021.745408 PMC8566722 34745176

[B59] Jiang G. Wei Z. Xu J. Chen H. Zhang Y. She X.. (2017). Bacterial wilt in China: history, current status, and future perspectives. Front. Plant Sci. 8. doi: 10.3389/fpls.2017.01549 PMC5601990 28955350

[B60] Jogi A. Kerry J. W. Brenneman T. B. Leebens-Mack J. H. Gold S. E. (2016). Identification of genes differentially expressed during early interactions between the stem rot fungus (Sclerotium rolfsii) and peanut (*Arachis hypogaea*) cultivars with increasing disease resistance levels. Microbiol. Res. 184, 1–12. doi: 10.1016/j.micres 26856448

[B61] Jones J. T. Haegeman A. Danchin E. G. Gaur H. S. Helder J. Jones M. G.. (2013). Top 10 plant-parasitic nematodes in molecular plant pathology. Mol. Plant Pathol. 14, 946–961. doi: 10.1111/mpp.12057 23809086 PMC6638764

[B62] Keshavareddy G. Rohini S. Ramu S. V. Sundaresha S. Kumar A. R. Kumar P. A.. (2013). Transgenics in groundnut (*Arachis hypogaea l.*) expressing cry1AcF gene for resistance to spodoptera litura (F.). Physiol. Mol. Biol. Plants 19, 343–352. doi: 10.1007/s12298-013-0182-6 24431503 PMC3715636

[B63] Kew M. C. (2013). Aflatoxins as a cause of hepatocellular carcinoma. j. gastrointestin. Liver Dis. 22, 305–310. doi: 10.1586/17474124.2013.832500 24078988

[B64] Khan S. A. Chen H. Deng Y. Chen Y. Zhang C. Cai T.. (2020). High-density SNP map facilitates fine mapping of QTLs and candidate genes discovery for aspergillus flavus resistance in peanut (*Arachis hypogaea*). Theor. Appl. Genet. 133, 2239–2257. doi: 10.1007/s00122-020-03594-0 32285164

[B65] Khedikar Y. P. Gowda M. V. Sarvamangala C. Patgar K. V. Upadhyaya H. D. Varshney R. K.. (2010). A QTL study on late leaf spot and rust revealed one major QTL for molecular breeding for rust resistance in groundnut (*Arachis hypogaea l.*). Theor. Appl. Genet. 121 (5), 971–984. doi: 10.1007/s00122-010-1366-x 20526757 PMC2921499

[B66] Khera P. Pandey M. K. Wang H. Feng S. Qiao L. Culbreath A. K.. (2016). Mapping quantitative trait loci of resistance to tomato spotted wilt virus and leaf spots in a recombinant inbred line population of peanut (*Arachis hypogaea l.*) from SunOleic 97R and NC94022. PloS One 11, e0158452. doi: 10.1371/journal.pone.0158452 27427980 PMC4948827

[B67] Kojima M. Kamada-Nobusada T. Komatsu H. Takei K. Kuroha T. Mizutani M.. (2009). Highly sensitive and high-throughput analysis of plant hormones using MS-probe modification and liquid chromatography-tandem mass spectrometry: an application for hormone profiling in oryza sativa. Plant Cell Physiol. 50, 1201–1214. doi: 10.1093/pcp/pcp057 19369275 PMC2709547

[B68] Kolekar R. M. Sukruth M. Shirasawa K. Nadaf H. L. Motagi B. N. Lingaraju S.. (2017). Marker-assisted backcrossing to develop foliar disease-resistant genotypes in TMV 2 variety of peanut (*Arachis hypogaea l*.). Plant Breed 136, 948–953. doi: 10.1111/pbr.12549

[B69] Krishna G. Singh B. K. Kim E. K. Morya V. K. Ramteke P. W. (2015). Progress in genetic engineering of peanut (*Arachis hypogaea l.*)–a review. Plant Biotechnol. J. 13, 147–162. doi: 10.1111/pbi.12339 25626474

[B70] Kumar R. Bohra A. Pandey A. K. Pandey M. K. Kumar A. (2017). Metabolomics for plant improvement: status and prospects. Front. Plant Sci. 8. doi: 10.3389/fpls.2017.01302 PMC5545584 28824660

[B71] Kumar K. R. Kirti P. B. (2011). Differential gene expression in *Arachis diogoi* upon interaction with peanut late leaf spot pathogen, phaeoisariopsis personata and characterization of a pathogen induced cyclophilin. Plant Mol. Biol. 75, 497–513. doi: 10.1007/s11103-011-9747-3 21298396

[B72] Kumar D. Kirti P. B. (2015). Transcriptomic and proteomic analyses of resistant host responses in *Arachis diogoi* challenged with late leaf spot pathogen, phaeoisariopsis personata. PloS One 10, e0117559. doi: 10.1371/journal.pone.0117559 25646800 PMC4315434

[B73] Kumar K. R. Srinivasan T. Kirti P. B. (2009). A mitogen-activated protein kinase gene, AhMPK3 of peanut: molecular cloning, genomic organization, and heterologous expression conferring resistance against spodoptera litura in tobacco. Mol. Genet. Genomics 282, 65–81. doi: 10.1007/s00438-009-0446-6 19352711

[B74] Le C. N. Mendes R. Kruijt M. Raaijmakers J. M. (2012). Genetic and phenotypic diversity of sclerotium rolfsii in groundnut felds in central Vietnam. Plant Dis. 96, 389–397. doi: 10.1094/PDIS-06-11-0468 30727129

[B75] Leal-Bertioli S. C. Cavalcante U. Gouvea E. G. Ballén-Taborda C. Shirasawa K. Guimarães P. M.. (2015). Identification of QTLs for rust resistance in the peanut wild species *Arachis magna* and the development of KASP markers for marker-assisted selection. G3 (Bethesda) 5, 1403–1413. doi: 10.1534/g3.115.018796 25943521 PMC4502374

[B76] Leal-Bertioli S. C. José A. C. Alves-Freitas D. M. Moretzsohn M. C. Guimarães P. M. Nielen S.. (2009). Identification of candidate genome regions controlling disease resistance in *Arachis* . BMC Plant Biol. 9, 112. doi: 10.1186/1471-2229-9-112 19698131 PMC2739205

[B77] Leal-Bertioli S. C. Moretzsohn M. C. Roberts P. A. Ballén-Taborda C. Borba T. C. Valdisser P. A.. (2016). Genetic mapping of resistance to meloidogyne arenaria in *Arachis stenosperma*: a new source of nematode resistance for peanut. G3 (Bethesda) 6, 377–390. doi: 10.1534/g3.115.023044 PMC4751557 26656152

[B78] Liang Y. Barring M. Wang S. Septiningsih E. M. (2017). Mapping QTLs for leafspot resistance in peanut using SNP-based next-generation sequencing markers. Plant Breed Biotech. 5, 115–122. doi: 10.9787/pbb.2017.5.2.115

[B79] Lister R. Gregory B. D. Ecker J. R. (2009). Next is now: new technologies for sequencing of genomes, transcriptomes, and beyond. Curr. Opin. Plant Biol. 12, 107–118. doi: 10.1016/j.pbi.2008.11.004 19157957 PMC2723731

[B80] Lu Q. Liu H. Hong Y. Li H. Liu H. Li X.. (2018). Consensus map integration and QTL meta-analysis narrowed a locus for yield traits to 0.7 cM and refined a region for late leaf spot resistance traits to 0.38 cM on linkage group A05 in peanut (*Arachis hypogaea l.*). BMC Genomics 19, 887. doi: 10.1186/s12864-018-5288-3 30526476 PMC6286586

[B81] Luo H. Pandey M. K. Khan A. W. Wu B. Guo J. Ren X.. (2019). Next-generation sequencing identified genomic region and diagnostic markers for resistance to bacterial wilt on chromosome B02 in peanut (*Arachis hypogaea l.*). Plant Biotechnol. J. 17, 2356–2369. doi: 10.1111/pbi.13153 31087470 PMC6835129

[B82] Luo H. Pandey M. K. Zhi Y. Zhang H. Xu S. Guo J.. (2020). Discovery of two novel and adjacent QTLs on chromosome B02 controlling resistance against bacterial wilt in peanut variety zhonghua 6. Theor. Appl. Genet. 133, 1133–1148. doi: 10.1007/s00122-020-03537-9 31980836 PMC7064456

[B83] Ma Y. Yang J. Yang D. Qin G. Zu J. (2022). Development of 1,5-Diaryl-Pyrazole-3-Formate analogs as antifungal pesticides and their application in controlling peanut stem rot disease. Front. Microbiol. 12, 728173. doi: 10.3389/fmicb.2021.728173 35058889 PMC8763808

[B84] Mahatma M. K. Thawait L. K. Jadon K. S. Thirumalaisamy P. P. Bishi S. K. Rathod K. J.. (2021). Metabolic profiling for dissection of late leaf spot disease resistance mechanism in groundnut. Physiol. Mol. Biol. Plants 27, 1027–1041. doi: 10.1007/s12298-021-00985-5 34108825 PMC8140181

[B85] Mansfield J. Genin S. Magori S. Citovsky V. Sriariyanum M. Ronald P.. (2012). Top 10 plant pathogenic bacteria in molecular plant pathology. Mol. Plant Pathol. 13, 614–629. doi: 10.1111/j.1364-3703.2012.00804.x 22672649 PMC6638704

[B86] Martins A. C. Q. Mehta A. Murad A. M. Mota A. P. Z. Saraiva M. A. P. Araújo A. C. G.. (2020). Proteomics unravels new candidate genes for meloidogyne resistance in wild *Arachis* . J. Proteomics 217, 103690. doi: 10.1016/j.jprot.2020 32068185

[B87] Mayee C. D. Datar V. V. Raychaudhuri S. P. Verma J. P. (1988). Diseases of groundnut in the tropics. Rev. Trop. Plant Pathol. 5, 85–118.

[B88] McDonald D. Subrahmanyam P. Gibbons R. W. Smith D. H. (1985). Early and late leaf spots of groundnut. Patancheru: ICRISAT Inf. Bulletin. 12, 1–19.

[B89] McGahan P. Lloyd R. J. Rynne K. P. (1991). “The cost of helicoverpa in Queensland crops,” in A review of heliothis research in Australia conference and workshop series QC91006. Eds. Twine H. Zalucki M. P. (Brisbane: Queensland Department of PrimaryIndustries), 11–28.

[B90] Mehan V. K. Mayee C. D. McDonald D. (1994). Management of sclerotium rolfsiicaused stem and pod rots of groundnut – a critical review. Int. J. Pest Manage 40, 313–320. doi: 10.1080/09670879409371906

[B91] Merwe P. Subrahmanyan P. Kimmins F. M. Willekens J. (2001). Mechanisms of resistance to groundnut rosette. Int. Arachis Newsl 21, 43–46.

[B92] Meyers B. C. Dickerman A. W. Michelmore R. W. Sivaramakrishnan S. Sobral B. W. Young N. D. (1999). Plant disease resistance genes encode members of an ancient and diverse protein family within the nucleotide-binding superfamily. Plant J. 20, 317–332. doi: 10.1046/j.1365-313x.1999.t01-1-00606.x 10571892

[B93] Middleton K. J. Saleh N. (1988). “Peanut stripe virus disease in Indonesia and the ACIAR project”. in in Coordination of Research on Peanut Stripe Virus. (Patancheru: ICRISAT), pp. 4–6.

[B94] Mochida K. Shinozaki K. (2010). Genomics and bioinformatics resources for crop improvement. Plant Cell Physiol. 51, 497–523. doi: 10.1093/pcp/pcq027 20208064 PMC2852516

[B95] Mondal S. Badigannavar A. M. (2018). Mapping of a dominant rust resistance gene revealed two r genes around the major Rust_QTL in cultivated peanut (*Arachis hypogaea l.*). Theor. Appl. Genet. 131, 1–11. doi: 10.1007/s00122-018-3106-6 29744525

[B96] Mondal S. Mohamed Shafi K. Raizada A. Song H. Badigannavar A. M. Sowdhamini R. (2022). Development of candidate gene-based markers and map-based cloning of a dominant rust resistance gene in cultivated groundnut (*Arachis hypogaea* l.). Gene 827, 146474. doi: 10.1016/j.gene.2022.146474 35390447

[B97] Moretzsohn M. C. Gouvea E. G. Inglis P. W. Leal-Bertioli S. C. Valls J. F. Bertioli D. J. (2013). A study of the relationships of cultivated peanut (*Arachis hypogaea*) and its most closely related wild species using intron sequences and microsatellite markers. Ann. Bot. 111, 113–126. doi: 10.1093/aob/mcs237 23131301 PMC3523650

[B98] Morgante C. V. Brasileiro A. C. M. Roberts P. A. Guimaraes L. A. Araujo A. C. G. Fonseca L. N.. (2013). A survey of genes involved in *Arachis stenosperma* resistance to meloidogyne arenaria race 1. Funct. Plant Biol. 40, 1298–1309. doi: 10.1071/FP13096 32481196

[B99] Mota A. P. Z. Fernandez D. Arraes F. B. M. Petitot A. S. de Melo B. P. de Sa M. E. L.. (2020). Evolutionarily conserved plant genes responsive to root-knot nematodes identified by comparative genomics. Mol. Genet. Genomics 295, 1063–1078. doi: 10.1007/s00438-020-01677-7 32333171

[B100] Mota A. P. Z. Vidigal B. Danchin E. G. J. Togawa R. C. Leal-Bertioli S. C. M. Bertioli D. J.. (2018). Comparative root transcriptome of wild *Arachis* reveals NBS-LRR genes related to nematode resistance. BMC Plant Bio l18, 159. doi: 10.1186/s12870-018-1373-7 PMC6080386 30081841

[B101] Nagy E. D. Chu Y. Guo Y. Khanal S. Tang S. Li Y.. (2010). Recombination is suppressed in an alien introgression in peanut harboring rma, a dominant root-knot nematode resistance gene. Mol. Breed. 26, 357–370. doi: 10.1007/s11032-010-9430-4

[B102] Nandini D. Mohan J. S. S. Singh G. (2010). Induction of systemic acquired resistance in *Arachis hypogaea l.* by sclerotium rolfsii derived elicitors. J. Phytopathol. 158, 594–600. doi: 10.1111/j.1439-0434.2009.01666.x

[B103] Okabe I. Matsumoto N. (2000). Population structure of sclerotium rolfsii in peanut fields. Mycoscience 41, 145–148. doi: 10.1007/BF02464323

[B104] Padgham D. E. Kimmins F. M. Rao G. V. R. (1990). Resistance in groundnut (*Arachis hypogaea* l.) to aphis craccivora (Koch). Ann. Appl. Biol. 117, 285–294. doi: 10.1111/j.1744-7348.1990.tb04214.x

[B105] Pandey M. K. Agarwal G. Kale S. M. Clevenger J. Nayak S. N. Sriswathi M.. (2017c). Development and evaluation of a high density genotyping 'Axiom_Arachis' array with 58 K SNPs for accelerating genetics and breeding in groundnut. Sci. Rep. 7, 40577. doi: 10.1038/srep40577 28091575 PMC5238394

[B106] Pandey M. K. Khan A. W. Singh V. K. Vishwakarma M. K. Shasidhar Y. Kumar V.. (2017b). QTL-seq approach identified genomic regions and diagnostic markers for rust and late leaf spot resistance in groundnut (*Arachis hypogaea l.*). Plant Biotechnol. J. 15, 927–941. doi: 10.1111/pbi.12686 28028892 PMC5506652

[B107] Pandey M. K. Kolekar R. M. Khedikar Y. P. Varshney (2016). QTL mapping for late leaf spot and rust resistance using an improved genetic map and extensive phenotypic data on a recombinant inbred line population in peanut (*Arachis hypogaea l.*). Euphytica Int. J. Plant Breed 209 (1), 147–156. doi: 10.1007/s10681-016-1651-0

[B108] Pandey M. K. Pandey A. K. Kumar R. Nwosu C. V. Guo B. Wright G. C.. (2020). Translational genomics for achieving higher genetic gains in groundnut. Theor. Appl. Genet. 133 (5), 1679–1702. doi: 10.1007/s00122-020-03592-2 32328677 PMC7214508

[B109] Pandey M. K. Wang H. Khera P. Vishwakarma M. K. Kale S. M. Culbreath A. K.. (2017a). Genetic dissection of novel QTLs for resistance to leaf spots and tomato spotted wilt virus in peanut (*Arachis hypogaea l.*). Front. Plant Sci. 8. doi: 10.3389/fpls.2017.00025 PMC5281592 28197153

[B110] Pappu H. R. Jones R. A. C. Jain R. K. (2009). Global status of tospovirus epidemics in diverse cropping systems: successes achieved and challenges ahead. Virus Res. 141, 219–236. doi: 10.1016/j.virusres.2009.01.009 19189852

[B111] Pasupuleti J. Pandey M. K. Manohar S. S. Variath M. T. Nallathambi P. Nadaf H. L.. (2016). Foliar fungal disease-resistant introgression lines of groundnut (*Arachis hypogaea l.*) record higher pod and haulm yield in multilocation testing. Plant Breed 135, 355–366. doi: 10.1111/pbr.12358

[B112] Peng W. F. Lv J. W. Ren X. P. Huang L. Zhao X. Y. Wen Q. G.. (2011). Differential expression of genes related to bacterial wilt resistance in peanut (*Arachis hypogaea l.*). Yi Chuan 33, 389–396. doi: 10.3724/sp.j.1005.2011.00389 21482530

[B113] Pérez-Clemente R. M. Vives V. Zandalinas S. I. López-Climent M. F. Muñoz V. Gómez-Cadenas A. (2013). Biotechnological approaches to study plant responses to stress. BioMed. Res. Int. 2013, 654120. doi: 10.1155/2013/654120 23509757 PMC3591138

[B114] Pittet A. (1998). Natural occurrence of mycotoxins in foods and feeds: an update review. Rev. Med. Vet. 149, 479–492. doi: 10.1016/j.fgb.2014.02.005

[B115] Prasad M. N. R. Gowda M. V. C. (2006). Mechanisms of resistance to tobacco cutworm (Spodoptera litura f.) and their implications to screening for resistance in groundnut. Euphytica 149, 387–399. doi: 10.1007/s10681-006-9088-5

[B116] Pratissoli D. Pirovani V. D. Lima W. L. (2015). Occurrence of helicoverpa armigera (Lepidoptera: noctuidae) on tomato in the espírito Santo state. Hortic. Bras. 33, 101–105. doi: 10.1590/S0102-053620150000100016

[B117] Proite K. Carneiro R. Falco R. Gomes A. Bertioli D. (2010). Post-infection development and histopathology of meloidogyne arenaria race 1 on *Arachis* spp. Plant Pathol. 57, 974–980. doi: 10.1111/j.1365-3059.2008.01861.x

[B118] Punja Z. K. (1985). The biology, ecology, and control of sclerotium rolfsii. Annu. Rev. Phytopathol. 23, 97–127. doi: 10.1146/annurev.py.23.090185.000525

[B119] Qi F. Sun Z. Liu H. Zheng Z. Qin L. Shi L.. (2022). QTL identification, fine mapping, and marker development for breeding peanut (*Arachis hypogaea l.*) resistant to bacterial wilt. Theor. Appl. Genet. 135, 1319–1330. doi: 10.1007/s00122-022-04033-y 35059781 PMC9033696

[B120] Qin H. Feng S. Chen C. Guo Y. Knapp S. Culbreath A.. (2012). An integrated genetic linkage map of cultivated peanut (*Arachis hypogaea l.*) constructed from two RIL populations. Theor. Appl. Genet. 124, 653–664. doi: 10.1007/s00122-011-1737-y 22072100

[B121] Rao S. C. Bhatnagar-Mathur P. Kumar P. L. Reddy A. S. Sharma K. K. (2013). Pathogen-derived resistance using a viral nucleocapsid gene confers only partial non-durable protection in peanut against peanut bud necrosis virus. Arch. Virol. 158, 133–143. doi: 10.1007/s00705-012-1483-8 23011312

[B122] Rathod V. Hamid R. Tomar R. S. Patel R. Padhiyar S. Kheni J.. (2020). Comparative RNA-seq profiling of a resistant and susceptible peanut (*Arachis* hypogaea) genotypes in response to leaf rust infection caused by puccinia arachidis. 3 Biotech. 10, 284. doi: 10.1007/s13205-020-02270-w PMC7266923 32550103

[B123] Ratnaparkhe M. B. Wang X. Li J. Compton R. O. Rainville L. K. Lemke C.. (2011). Comparative analysis of peanut NBS-LRR gene clusters suggests evolutionary innovation among duplicated domains and erosion of gene microsynteny. New Phytol. 192, 164–178. doi: 10.1111/j.1469-8137.2011.03800.x 21707619

[B124] Riley D. G. Joseph S. V. Srinivasan R. Diffiffiffie S. (2011). Thrips vectors of tospoviruses. J. Integ. Pest. Manage. 2, 1–10. doi: 10.1603/IPM10020

[B125] Saito K. Matsuda F. (2010). Metabolomics for functional genomics, systems biology, and biotechnology. Annu. Rev. Plant Biol. 61, 463–489. doi: 10.1146/annurev.arplant.043008.092035 19152489

[B126] Salanoubat M. Genin S. Artiguenave F. Gouzy J. Mangenot S. Arlat M.. (2002). Genome sequence of the plant pathogen ralstonia solanacearum. Nature 415, 497–502. doi: 10.1038/415497a 11823852

[B127] Saleh N. Horn N. M. Reddy D. V. R. Middleton K. J. (1989). Peanut stripe virus in Indonesia. Eur. J. Plant Pathol. 99, 123–127. doi: 10.1007/bf01997480

[B128] Sharma H. C. (2005). Heliothis/Helicoverpa management: emerging trends and strategies for future research (New Delhi, India: Oxford and IBH Publishing Co.Pvt. Ltd), 469 pp.

[B129] Sharma S. Choudhary B. Yadav S. Mishra A. Mishra V. K. Chand R.. (2021). Metabolite profiling identified pipecolic acid as an important component of peanut seed resistance against aspergillus flavus infection. J. Hazard Mater 404, 124155. doi: 10.1016/j.jhazmat.2020.124155 33049626

[B130] Sharma H. C. Pampapathy G. Dwivedi S. L. Reddy L. J. (2003). Mechanisms and diversity of resistance to insect pests in wild relatives of groundnut. J. Econ Entomol 96, 1886–1897. doi: 10.1093/jee/96.6.1886 14977130

[B131] Shasidhar Y. Variath M. T. Vishwakarma M. K. Manohar S. S. Gangurde S. S. Sriswathi M.. (2020). Improvement of three popular Indian groundnut varieties for foliar disease resistance and high oleic acid using SSR markers and SNP array in marker-assisted backcrossing. Crop J. 8, 1–15.

[B132] Shinozaki K. Sakakibara H. (2009). Omics and bioinformatics: an essential toolbox for systems analyses of plant functions beyond 2010. Plant Cell Physiol. 50, 1177–1180. doi: 10.1093/pcp/pcp085 19596708 PMC2709552

[B133] Shirasawa K. Bhat R. S. Khedikar Y. P. Sujay V. Kolekar R. M. Yeri S. B.. (2018). Sequencing analysis of genetic loci for resistance for late leaf spot and rust in peanut (*Arachis hypogaea l.*). Front. Plant Sci. 9. doi: 10.3389/fpls.2018.01727 PMC6275244 30534132

[B134] Simpson C. E. Starr J. L. (2001). Registration of ‘COAN’ peanut. Crop Sci. 41, 918–918. doi: 10.2135/cropsci2001.413918x

[B135] Simpson C. E. Starr J. L. Church G. T. Burow M. D. Paterson A. H. (2003). Registration of ‘NemaTAM’ peanut. Crop Sci. 43, 1561–1561. doi: 10.2135/cropsci2003.1561

[B136] Singh M. K. Chandel V. Hallan V. Ram R. Zaidi A. A. (2009). Occurrence of peanut stripe virus on patchouli and raising of virus-free patchouli plants by meristem tip culture. J. Plant Dis. Prot 116, 2–6. doi: 10.1007/BF03356278

[B137] Song H. Wang P. Li C. Han S. Zhao C. Xia H.. (2017). Comparative analysis of NBS-LRR genes and their response to aspergillus flavus in *Arachis* . PloS One 12, e0171181. doi: 10.1371/journal.pone.0171181 28158222 PMC5291535

[B138] Srinivasa, Rao. M. Manimanjari D. Vanaja M. Rama, Rao. C. A. Srinivas K. Rao V. U.. (2012). Impact of elevated CO₂ on tobacco caterpillar, spodoptera litura on peanut, *Arachis hypogea* . J. Insect Sci. 12, 103. doi: 10.1673/031.012.10301 23437971 PMC3605029

[B139] Stalker H. T. Beute M. K. Shew B. B. Barker K. R. (2002). Registration of two root-knot nematode-resistant peanut germplasm lines. Crop Sci. 42, 312–313. doi: 10.2135/cropsci2002.312a 11756306

[B140] Stalker H. T. Campbell W. V. (1983). Resistance of wild species of peanut to an insect complex. Peanut Sci. 10, 30–33. doi: 10.3146/i0095-3679-10-1-9

[B141] Subrahmanyam P. McDonald. D. Reddy. U. Nigam. S. N. Smith. D. H. (1993). Origin and utilization of rust resistance in groundnut. Curr. Plant Sci. Biotechnol. Agr. 18, 147–158. doi: 10.1007/978-94-011-2004-3_12

[B142] Sujay V. Gowda M. V. Pandey M. K. Bhat R. S. Khedikar Y. P. Nadaf H. L.. (2012). Quantitative trait locus analysis and construction of consensus genetic map for foliar disease resistance based on two recombinant inbred line populations in cultivated groundnut (*Arachis hypogaea l.*). Mol. Breed 30 (2), 773–788. doi: 10.1007/s11032-011-9661-z 22924018 PMC3410029

[B143] Tay W. T. Soria M. F. Walsh T. Thomazoni D. Silvie P. Behere G. T.. (2013). A brave new world for an old world pest: helicoverpa armigera (Lepidoptera: noctuidae) in Brazil. PloS One 8, e80134. doi: 10.1371/journal.pone.0080134 24260345 PMC3832445

[B144] Termorshuizen A. J. (2007). Fungal and fungus-like pathogens of potato. Potato Biol. Biotechnol., 643–665. doi: 10.1016/b978-044451018-1/50071-3

[B145] Thiessen L. D. Woodward J. E. (2012). Diseases of peanut caused by soilborne pathogens in the southwestern united states. ISRN Agron., 517905. doi: 10.5402/2012/517905

[B146] Tirumalaraju S. V. Jain M. Gallo M. (2011). Differential gene expression in roots of nematode-resistant and -susceptible peanut (*Arachis hypogaea*) cultivars in response to early stages of peanut root-knot nematode (Meloidogyne arenaria) parasitization. J. Plant Physiol. 168, 481–492. doi: 10.1016/j.jplph.2010.08.006 20863592

[B147] Torres A. M. Barros G. G. Palacios S. A. Chulze S. N. Battilani P. (2014). Review on pre and post-harvest management of peanuts to minimize aflatoxin contamination. Food Res. Int. 62, 11–19. doi: 10.1016/j.foodres.2014.02.023

[B148] Trudgill D. L. Blok V. C. (2001). Apomictic, polyphagous root-knot nematodes: exceptionally successful and damaging biotrophic root pathogens. Annu. Rev. Phytopathol. 39, 53–77. doi: 10.1146/annurev.phyto.39.1.53 11701859

[B149] Tseng Y. C. Tillman B. L. Peng Z. Wang J. (2016). Identifcation of major QTLs underlying tomato spotted wilt virus resistance in peanut cultivar Florida-EP ‘113’. BMC Genet. 17, 128. doi: 10.1186/s12863-016-0435-9 27600750 PMC5012072

[B150] Tshilenge-Lukanda L. Nkongolo K. K. C. Kalonji-Mbuyi A. Kizungu R. V. (2012). Epidemiology of the groundnut (*Arachis hypogaea l.*) leaf spot disease: genetic analysis and developmental cycles. Am. J. Plant Sci. 3, 582–588. doi: 10.4236/ajps.2012.35070

[B151] Urcuqui-Inchima S. Haenni A. L. Bernardi F. (2001). Potyvirus proteins: a wealth of functions. Virus Res. 74, 157–175. doi: 10.1016/S0168-1702(01)00220-9 11226583

[B152] Vadivel A. K. (2015). Gel-based proteomics in plants: time to move on from the tradition. front. Plant Sci. 6. doi: 10.3389/fpls.2015.00369 PMC4470439 26136753

[B153] Varshney R. K. Pandey M. K. Bohra A. Singh V. K. Thudi M. Saxena R. K. (2019). Toward the sequence-based breeding in legumes in the post-genome sequencing era. Theor. Appl. Genet. 132 (3), 797–816. doi: 10.1007/s00122-018-3252-x 30560464 PMC6439141

[B154] Varshney R. K. Pandey M. K. Janila P. Nigam S. N. Sudini H. Gowda M. V.. (2014). Marker-assisted introgression of a QTL region to improve rust resistance in three elite and popular varieties of peanut (*Arachis hypogaea l.*). Theor. Appl. Genet. 127, 1771–1781. doi: 10.1007/2Fs00122-014-2338-3 24927821 PMC4110420

[B155] Wang Z. Huai D. Zhang Z. Cheng K. Kang Y. Wan L.. (2018). Development of a high-density genetic map based on specific length amplified fragment sequencing and its application in quantitative trait loci analysis for yield-related traits in cultivated peanut. Front. Plant Sci. 9. doi: 10.3389/fpls.2018.00827 PMC6028809 29997635

[B156] Wang H. Lei Y. Wan L. Yan L. Lv J. Dai X.. (2016). Comparative transcript profiling of resistant and susceptible peanut post-harvest seeds in response to aflatoxin production by aspergillus flavus. BMC Plant Biol. 16, 54. doi: 10.1186/s12870-016-0738-z 26922489 PMC4769821

[B157] Wang H. Penmetsa R. V. Yuan M. Gong L. Zhao Y. Guo B.. (2012). Development and characterization of BAC-end sequence derived SSRs, and their incorporation into a new higher density genetic map for cultivated peanut (*Arachis hypogaea l.*). BMC Plant Biol. 12, 10. doi: 10.1186/1471-2229-12-10 22260238 PMC3298471

[B158] Wang T. Zhang E. Chen X. Li L. Liang X. (2010). Identification of seed proteins associated with resistance to pre-harvested aflatoxin contamination in peanut (*Arachis hypogaea l.*). BMC Plant Biol. 10, 267. doi: 10.1186/1471-2229-10-267 21118527 PMC3095339

[B159] War A. R. Paulraj M. G. War M. Y. Ignacimuthu S. (2011). Jasmonic acid-mediated-induced resistance in groundnut (*Arachis hypogaea l.*) against helicoverpa armigera (hubner) (lepidoptera: noctuidae). J. Plant Growth Regul. 30, 512–523. doi: 10.1007/s00344-011-9213-0

[B160] War A. R. Sharma S. P. Sharma H. C. (2016). Differential induction of flavonoids in groundnut in response to helicoverpa armigera and aphis craccivora infestation. Int. J. Insect Sci. 8, 55–64. doi: 10.4137/IJIS.S39619 27398031 PMC4933539

[B161] Wei T. Zhang C. Hong J. Xiong R. Kasschau K. D. Zhou X.. (2010). Formation of complexes at plasmodesmata for potyvirus intercellular movement is mediated by the viral protein P3N-PIPO. PloS Pathog. 6, e1000962. doi: 10.1371/journal.ppat.1000962 20585568 PMC2891837

[B162] Wightman J. A. Rao G. V. (1994). Groundnut pests. World Crop, 395–479. doi: 10.1007/978-94-011-0733-4_11

[B163] Xu Z. Y. (2002). Progress on research to control peanut virus disease. Plant Protect. Technol. Ext. 22, 37–39.

[B164] Xu Z. Y. Chen K. R. Zhang Z. Y. Chen J. X. (1991). Seed transmission of peanut stripe virus in peanut. Plant Dis. 75, 723–726. doi: 10.1094/PD-75-0723

[B165] Xu Z. Y. Yu Z. Liu J. L. Barnett O. W. (1983). A virus causing peanut mild mottle in hubei province, China. Plant Dis. 67, 1029–1032. doi: 10.1094/PD-67-1029

[B166] Xu P. Li H. Wang X. Zhao G. Lu X. Dai S.. (2022). Integrated analysis of the lncRNA/circRNA-miRNA-mRNA expression profiles reveals novel insights into potential mechanisms in response to root-knot nematodes in peanut. BMC Genomics 23, 239. doi: 10.1186/s12864-022-08470-3 35346027 PMC8962500

[B167] Xu M. Xie H. Wu J. Xie L. Yang J. Chi Y. (2017). Translation initiation factor eIF4E and eIFiso4E are both required for peanut stripe virus infection in peanut (*Arachis hypogaea l.*). Front. Microbiol. 8. doi: 10.3389/fmicb.2017.00338 PMC5344889 28344571

[B168] Yan L. Jin H. Raza A. Huang Y. Gu D. Zou X. (2022). WRKY genes provide novel insights into their role against ralstonia solanacearum infection in cultivated peanut (*Arachis hypogaea l.*). Front. Plant Sci. 13. doi: 10.3389/fpls.2022.986673 PMC9531958 36204053

[B169] Yan L. Wang Z. Song W. Fan P. Kang Y. Lei Y.. (2021). Genome sequencing and comparative genomic analysis of highly and weakly aggressive strains of sclerotium rolfsii, the causal agent of peanut stem rot. BMC Genomics 22, 276. doi: 10.1186/s12864-021-07534-0 33863285 PMC8052761

[B170] Yu B. Huai D. Huang L. Kang Y. Ren X. Chen Y.. (2019). Identification of genomic regions and diagnostic markers for resistance to aflatoxin contamination in peanut (*Arachis hypogaea l.*). BMC Genet. 20, 32. doi: 10.1186/s12863-019-0734-z 30866805 PMC6417274

[B171] Yuksel B. Estill J. Schulze S. Paterson A. (2005). Organization and evolution of resistance gene analogs in peanut. Mol. Gen. Genomics 274, 248–263. doi: 10.1007/s00438-005-0022-7 16179993

[B172] Zhang C. Chen H. Cai T. Deng Y. Zhuang R. Zhang N.. (2017). Overexpression of a novel peanut NBS-LRR gene AhRRS5 enhances disease resistance to ralstonia solanacearum in tobacco. Plant Biotechnol. J. 15, 39–55. doi: 10.1111/pbi.12589 27311738 PMC5253469

[B173] Zhang C. Chen H. Zhuang R. R. Chen Y. T. Deng Y. Cai T. C.. (2019). Overexpression of the peanut CLAVATA1-like leucine-rich repeat receptor-like kinase AhRLK1 confers increased resistance to bacterial wilt in tobacco. J. Exp. Bot. 70, 5407–5421. doi: 10.1093/jxb/erz274 31173088 PMC6793444

[B174] Zhang H. Chu Y. Dang P. Tang Y. Jiang T. Clevenger J. P.. (2020). Identifcation of QTLs for resistance to leaf spots in cultivated peanut (*Arachis hypogaea l.*) through GWAS analysis. Theor. Appl. Genet. 133, 2051–2061. doi: 10.1007/s00122-020-03576-2 32144466

[B175] Zhang W. Xian J. Sun C. Wang C. Shi L. Yu W. (2021). Preliminary study of genome editing of peanut FAD2 genes by CRISPR/Cas9. Acta Agronomica Sin. 47, 1481–1490.

[B176] Zhao X. Li C. Yan C. Wang J. Shan S. (2019). Transcriptome and proteome analyses of resistant preharvest peanut seed coat in response to aspergillus flavus infection. Electron J. Biotechn 39, 82–90. doi: 10.1016/j.ejbt.2019.03.003

[B177] Zhao C. Li T. Zhao Y. Zhang B. Li A. Zhao S.. (2020). Integrated small RNA and mRNA expression profiles reveal miRNAs and their target genes in response to aspergillus flavus growth in peanut seeds. BMC Plant Biol. 20, 215. doi: 10.1186/s12870-020-02426-z 32404101 PMC7222326

[B178] Zhao C. Qiu J. Agarwal G. Wang J. Ren X. Xia H.. (2017). Genome-wide discovery of microsatellite markers from diploid progenitor species, *Arachis duranensis* and *A. ipaensis*, and their application in cultivated peanut (*A. hypogaea*). Front. Plant Sci. 8. doi: 10.3389/fpls.2017.01209 PMC5513918 28769940

[B179] Zhao Z. Tseng Y. C. Peng Z. Lopez Y. Chen C. Y. Tillman B. L.. (2018). Refining a major QTL controlling spotted wilt disease resistance in cultivated peanut (*Arachis hypogaea l.*) and evaluating its contribution to the resistance variations in peanut germplasm. BMC Genet. 19, 17. doi: 10.1186/s12863-018-0601-3 29571286 PMC5865372

[B180] Zhao Y. Zhang C. Chen H. Yuan M. Nipper R. Prakash C. S.. (2016). QTL mapping for bacterial wilt resistance in peanut (*Arachis hypogaea l.*). Mol. Breed 36, 13. doi: 10.1007/s11032-015-0432-0 26869849 PMC4735223

[B181] Zhou X. Xia Y. Liao J. Liu K. Li Q. Dong Y.. (2016). Quantitative trait locus analysis of late leaf spot resistance and plant-Type-Related traits in cultivated peanut (*Arachis hypogaea l.*) under multi-environments. PloS One 11, e0166873. doi: 10.1371/journal.pone.0166873 27870916 PMC5117734

[B182] Zhuang W. Chen H. Yang M. Wang J. Pandey M. K. Zhang C.. (2019). The genome of cultivated peanut provides insight into legume karyotypes, polyploid evolution and crop domestication. Nat. Genet. 51 (5), 865–876. doi: 10.1038/s41588-019-0402-2 31043757 PMC7188672

